# Gestational Exposure to Phthalates and Phthalate Replacements in Relation to Neurodevelopmental Delays in Early Childhood

**DOI:** 10.3390/toxics11010065

**Published:** 2023-01-11

**Authors:** Seonyoung Park, Emily Zimmerman, Gredia Huerta-Montañez, Zaira Rosario-Pabón, Carmen M. Vélez-Vega, José F. Cordero, Akram Alshwabekah, John D. Meeker, Deborah J. Watkins

**Affiliations:** 1 Department of Environmental Health Sciences, University of Michigan School of Public Health, Ann Arbor, MI 48109, USA; 2 Department of Communication Sciences and Disorders, Northeastern University, Boston, MA 02115, USA; 3 Department of Electrical and Computer Engineering, Northeastern University, Boston, MA 02115, USA; 4 Department of Social Sciences, UPR Medical Sciences Campus, University of Puerto Rico Graduate School of Public Health, San Juan, PR 00936, USA; 5 Department of Epidemiology and Biostatistics, University of Georgia, Athens, GA 30602, USA; 6 Department of Civil and Environmental Engineering, Northeastern University, Boston, MA 02115, USA

**Keywords:** phthalates, phthalate replacements, neurodevelopment, sex-specificity

## Abstract

Phthalates have been linked to changes in child neurodevelopment. However, sex-specificity has been reported inconsistently, and little is known about the impact of recent phthalate replacement chemicals. Our analysis included mother–child pairs (N = 274) from the PROTECT birth cohort in Puerto Rico. Phthalate metabolites were measured in multiple maternal urine collected during pregnancy. Neurodevelopment was measured at 6, 12, and 24 months of age using the Battelle Developmental Inventory-2nd edition (BDI), which provides scores for adaptive, personal-social, communication, motor, and cognitive domains. Multivariable linear regression was used to examine associations between phthalate metabolite concentrations and BDI scores, adjusting for maternal age, maternal education, child age, and specific gravity. Sex-specificity was assessed with sex X exposure interaction terms and stratified models. Results show that all five domains were significantly associated with mono-3-carboxypropyl phthalate (MCPP) at age 24 months, suggesting a holistic developmental delay related to this metabolite. Sex-specificity existed for all timepoints (p-interaction < 0.2), in general, showing stronger associations among boys. For example, metabolites of a recent phthalate replacement, di-2-ethylhexyl terephthalate (DEHTP), were differentially associated with the adaptive domain (boys −7.53%/IQR, 95% CI: −14.58, −0.48 vs. girls −0.85%/IQR, 95% CI: −5.08, 3.37), and the cognitive domain (boys −6.05%/IQR, 95% CI: −10.88, −1.22 vs. girls −1.93%/IQR, 95%CI: −4.14, 0.28) at 6 months. To conclude, gestational exposure to phthalates and phthalate replacements was associated with neurodevelopmental delay across multiple domains, with differences by sex and child age.

## 1. Introduction

Child neurodevelopmental delay refers to a delay in one or more neurodevelopmental domains—cognitive, adaptive, language, and motor skills [1]. The risk of neurodevelopmental delay in the United States was estimated at 4.7% among children of three to seven years of age between 2014 and 2016, even when excluding serious clinical disorders such as autism spectrum disorder or intellectual disability [2]. Early life neurodevelopmental delay is significant, not only because it contributes to rising health care costs and productivity costs, but because such a delay may evolve as a child grows [3]. Gestation is a particularly vulnerable period for environmental exposure because endocrine-disrupting chemicals can create an imbalance in hormones that play an important role in development, generate maternal oxidative stress, and dysregulate immune responses which may compromise fetal development. Alternatively, neurotoxic chemicals may directly affect the fetus by crossing the placenta, and the impacts may last until later in life. Establishing linkages between environmental exposures and neurodevelopmental delays will have great public health significance as many exposures can be modifiable through new interventions and policies at the local and federal levels.

Phthalates are chemicals widely used in the production of plastics and personal care products, in addition to many other applications. Thus, exposure to phthalates is ubiquitous in the general population. Phthalates may disrupt the thyroid signaling pathway [4], induce intracellular oxidant/antioxidant imbalance [5], break double-stranded DNA in human neurons [6], or alter brain structure [7], which may ultimately negatively affect fetal growth and brain development. In addition, phthalates are known to be estrogenic and anti-androgenic [8,9,10,11]. Studies have shown that phthalate exposures during gestation are associated with altered sex hormone levels of mothers [12,13,14,15,16], which have been linked to neurodevelopmental problems in the offspring [17,18,19].

Epidemiologic studies suggest that intrauterine phthalate exposure is linked to neurodevelopmental delay in early childhood (one year to school age), including cognitive and behavioral function [20,21,22,23]. However, findings are not fully conclusive and limited overlap exists across the neurodevelopmental domains assessed. In addition, developmental delay in early infancy in relation to phthalate exposure is not well understood, and inconsistent sex-specificity has been reported. Furthermore, the health effects of phthalate replacements such as di-2-ethylhexyl terephthalate (DEHTP) on child neurodevelopmental delay have not been addressed in observational studies even as exposure to these chemicals is increasing in the US [24].

To fill this knowledge gap, our study aimed to examine relationships between biomarkers of gestational exposure to phthalates, including a recent phthalate replacement chemical, and neurodevelopment across five domains (adaptive, personal-social, communication, motor, and cognitive domains) in children at 6, 12, or 24 months of age in Puerto Rico. We hypothesized that prenatal exposure to phthalates or phthalate replacements is associated with child neurodevelopmental delay from 6 to 24 months of age, and that sex-specificity exists in such relationships.

## 2. Materials and Methods

### 2.1. Study Participants

**Figure 1:**
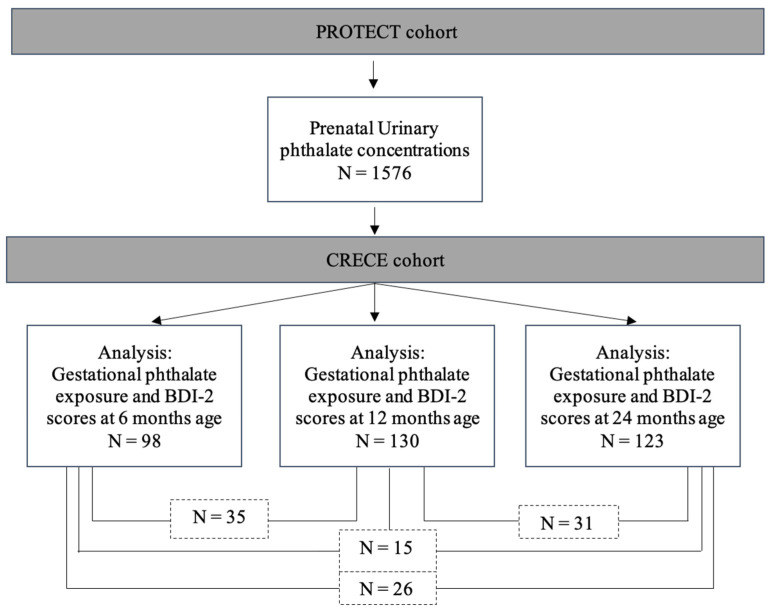
Analysis sample sizes. The present analysis included 274 participants with prenatal phthalate metabolite concentrations and childhood neurobehavioral assessments from at least one timepoint (age 6 months, 12 months, and/or 24 months). The dashed boxes below each analysis sample represent the number of participants with repeated assessment results.

Participants were part of the ongoing prospective birth cohort, the PROTECT study in Puerto Rico, which was initiated in 2010. Women were recruited at approximately 14 ± 2 weeks of gestation and inclusion criteria were that they (1) were between 18 and 40 years old, (2) lived in the Northern karst region, (3) did not use oral contraceptives three months prior to pregnancy, (4) did not report major obstetrical or medical complications, and (5) did not use in vitro fertilization to achieve the pregnancy [25]. Prenatal urine samples were collected during study visits at approximately 18, 22, and 26 weeks of gestation, and demographic and socioeconomic information was collected through a series of detailed questionnaires [26]. At the time of this analysis, a total of 1576 PROTECT women had at least one prenatal phthalate metabolite measurement (Figure 1). The outcome measures of child neurodevelopment were collected by the Center for Research on Early Childhood Exposure and Development in Puerto Rico (CRECE), which began in 2016 to track children who were born to PROTECT mothers through the age of four years [27]. Among the PROTECT participants who (1) met the CRECE recruitment criteria, and (2) were still younger than four years old, 555 babies were enrolled in the CRECE cohort and followed up at multiple time points, including 6–8 months, 12 months, and 24 months. In this analysis, 274 children with neurobehavioral assessments from at least one timepoint were included. Fifteen children had assessments at all three timepoints, and 77 had assessments at two timepoints (Figure 1).

This study was approved by the research and ethics committees of the University of Michigan School of Public Health, Northeastern University, the University of Puerto Rico, and participating hospitals and clinics. Study participants received a full description of the study and provided full informed consent prior to participation.

### 2.2. Phthalate Metabolite Measurements

Phthalate metabolites were measured in maternal urine samples collected during each prenatal study visit. All samples were frozen at −80 °C and shipped on dry ice to the Center for Disease Control and Prevention for analysis. Urinary concentrations of 15 phthalate and two DEHTP metabolites were measured using solid-phase extraction HPLC-isotope dilution tandem mass spectrometry as previously described [28]. Measured phthalate metabolites were mono(2-ethylhexyl) phthalate (MEHP), mono(2-ethyl-5-hydroxyhexyl) phthalate (MEHHP), mono(2-ethyl-5-oxohexyl) phthalate (MEOHP), mono(2-ethyl-5-carboxypentyl) phthalate (MECPP), mono-benzyl phthalate (MBzP), monocarboxyoctyl phthalate (MCOP), mono-isononyl phthalate (MNP), mono-oxoisononyl phthalate (MONP), monocarboxynonyl phthalate (MCNP), mono (3-carboxypropyl) phthalate (MCPP), mono-ethyl phthalate (MEP), mono-n-butyl phthalate (MBP), mono-3-hydroxybutyl phthalate (MHBP), mono- isobutyl phthalate (MiBP) and mono-2-hydroxy-iso-butyl phthalate (MHiBP) and two DEHTP metabolites, mono-2-ethyl-5-carboxypentyl terephthalate (MECPTP) and mono-2-ethyl-5-hydroxyhexyl terephthalate (MEHHTP) [29]. Summary measures of phthalate parent compounds were calculated by adding the molar fractions of metabolites; MEHP, MEHHP, MEOHP and MECPP for ΣDEHP; MBP and MHBP for ΣDBP; MiBP and MHiBP for ΣDiBP; MECPTP and MEHHTP for ΣDEHTP. We then multiplied summary measures by the molecular weight of the primary metabolite for unit comparability (ng/mL); MEHP (278.348 g/mol) for ΣDEHP; MBP (222.24 g/mol) for ΣDBP; MiBP (222.24 g/mol) for ΣDiBP; MEHHTP (294.34 g/mol) for ΣDEHTP, as previously described [30]. Values below the limit of detection (LOD) were substituted by LOD divided by the square root of 2. This imputation method has been shown to produce nonbiased means and SDs when the percentage of samples below the LOD is low, and is justified because all phthalate metabolites in our analysis were detected in more than 70% of urine samples collected across the study visits [31]. This approach has also been applied in other studies leveraging urinary phthalate metabolite biomarkers [32,33,34]. To account for urinary dilution, specific gravity (SG) was measured using a handheld digital refractometer (Atago Co., Ltd., Tokyo, Japan) at the University of Puerto Rico Medical Sciences Campus at the time of the sample collection. Urinary concentrations were corrected for specific gravity using the following formula: Pc = P [SGm-1SGi-1], where Pc is the specific gravity-corrected concentration (ng/mL), P is the measured urinary concentration, SGm is the median specific gravity across the study population (1.019), and SGi is the specific gravity for each participant [28]. Previous studies show that one urinary phthalate measurement reflects very recent exposure, whereas averaging multiple repeated urinary measurements is a better characterization of exposure over time [30]. Therefore, geometric means of specific gravity-corrected phthalate metabolite levels across pregnancy were used in the analysis.

### 2.3. Neurobehavioral Assessments

We used the Battelle Developmental Index-2 Spanish edition (BDI-2) to evaluate children’s neurodevelopment at the age of 6 months, 12 months, or 24 months. The BDI-2 assesses age-specific neurobehavioral function across five domains—adaptive, cognitive, personal-social, communication, and motor—using structured, observational, and interview items [35].

Each domain consists of one or two “subdomains” and there are nine subdomains in total—self-care under the adaptive domain (ADP); adult interaction, and self-concept and social role under personal-social domain (PS); receptive communication and expressive communication under communication domain (COM); gross motor, and fine motor under motor domain (MOT); attention and memory, reasoning and academic skills, and perception and concepts under cognitive domain (COG) [35,36]. The BDI-2 yields a developmental quotient (DQ) score for each domain and a scaled score for each subdomain, and both the DQ score and scaled score are norm-based (mean of 100, SD of 15 for domain scores and mean of 10, SD of 3 for the subdomain). Lower DQ scores or scaled scores indicate a poorer performance in the corresponding domain or subdomain. BDI-2 total scores, composites of all the domain test information, were calculated by summing the scaled scores of all subdomains within each domain. Five domain DQ scores and nine subdomain scaled scores were used in the analysis, and scores at the three-time points were used separately to examine the association with prenatal phthalate exposures.

### 2.4. Statistical Analyses

Distributions of urinary exposure biomarkers and BDI-2 outcome measures were assessed prior to analysis. Urinary phthalate metabolite concentrations were natural log-transformed and BDI-2 scores were added as a continuous variable in regression models. The associations between urinary concentrations of phthalate and replacement metabolites and BDI-2 domain DQ scores and subdomain scaled scores were examined using multivariable regression models, adjusting for potential confounders. Separate models were used for each phthalate metabolite and each BDI-2 domain or subdomain score was measured at each time point. Additionally, the role of the child’s sex as an effect modifier was assessed by adding interaction terms of sex and urinary phthalate concentration in regression models. If evidence of an interaction between phthalate metabolites and sex on the BDI-2 score was observed (*p*-value for interaction term < 0.2), sex-stratified models were used to investigate the sex-dependency of the association of interest. Covariates were selected based on a priori knowledge and impact on the main effect estimate (≥10%). The covariates considered were maternal age, maternal education, pre-pregnancy BMI, marital status, environmental tobacco smoke, alcohol consumption, and number of children. The final models were adjusted for maternal age (continuous), maternal education (categorical; GED or less, some college, Bachelors or higher), child age in months, and child sex (for non-stratified models). Regression results are presented as the percentage difference in score (95% confidence interval) per interquartile range (IQR) increase in urinary phthalate metabolite levels.

## 3. Results

### 3.1. Participant Characteristics

**Figure 2:**
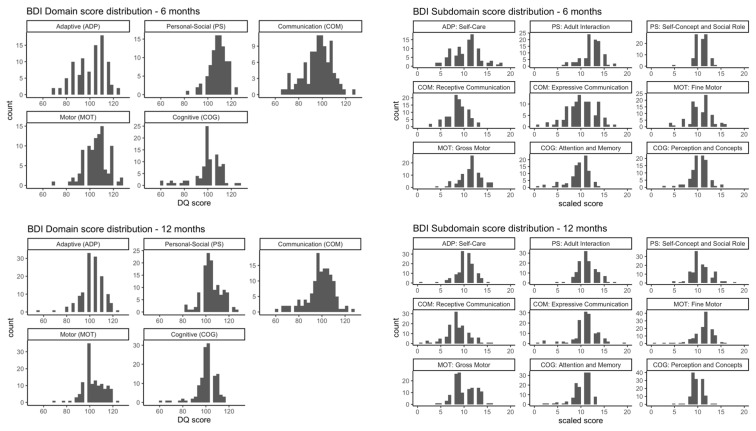

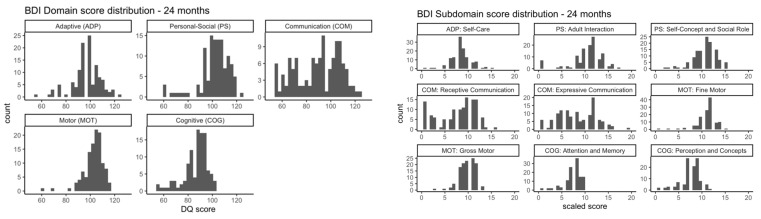
Histogram of BDI-2 domain and subdomain scores at 6 months, 12 months, and 24 months. Domain abbreviations: Adaptive (ADP), Personal-Social (PS), Communication (COM), Motor (MOT), and Cognitive (COG).

Table 1 summarizes the demographic characteristics of mothers and newborns, with characteristics stratified by infant sex and age at assessment in Supplementary Table S1. The present analysis includes 274 participants with prenatal phthalate and childhood outcome measures at the age of 6 months (n = 98), 12 months (n = 130), or 24 months (n = 123) (Figure 1). The distribution of BDI-2 domain/subdomain scores are presented in Figure 2 and Supplementary Table S2. Briefly, the means of BDI-2 total scores were within the normal range (population mean ± 1 SD) for all age points (6 months; 102.8 ± 8.8, 12 months; 94.9 ± 12.3, and 24 months; 103.5 ± 9.7) [36]. More children had communication domain DQ scores 2SD below the population mean (n = 21, 17%) in the 24 months sample compared to the other time points (Supplementary Figure S2). Most measured phthalate metabolites were detected in greater than 80 % of prenatal urine samples as previously reported [37,38]. The distributions of maternal urinary phthalate metabolite levels over the gestation are presented in Supplementary Figure S1 and Supplementary Table S3. The geometric mean of phthalate metabolite levels and distribution in our study population is similar to the previously reported distribution in the larger PROTECT population, with some phthalates higher than NHANES 2011–2016 [30]. We did not observe any significant difference in urinary phthalate metabolite levels between study visits based on a one-way ANOVA test (Supplementary Table S3). Similarly, there was no significant difference in prenatal phthalate metabolite levels between children with assessments at the different age time points (6 months, 12 months, or 24 months) except for DBP (ANOVA, *p* > 0.05).

### 3.2. Intrauterine Phthalate Exposure and BDI-2 in Early Childhood

**Figure 3:**
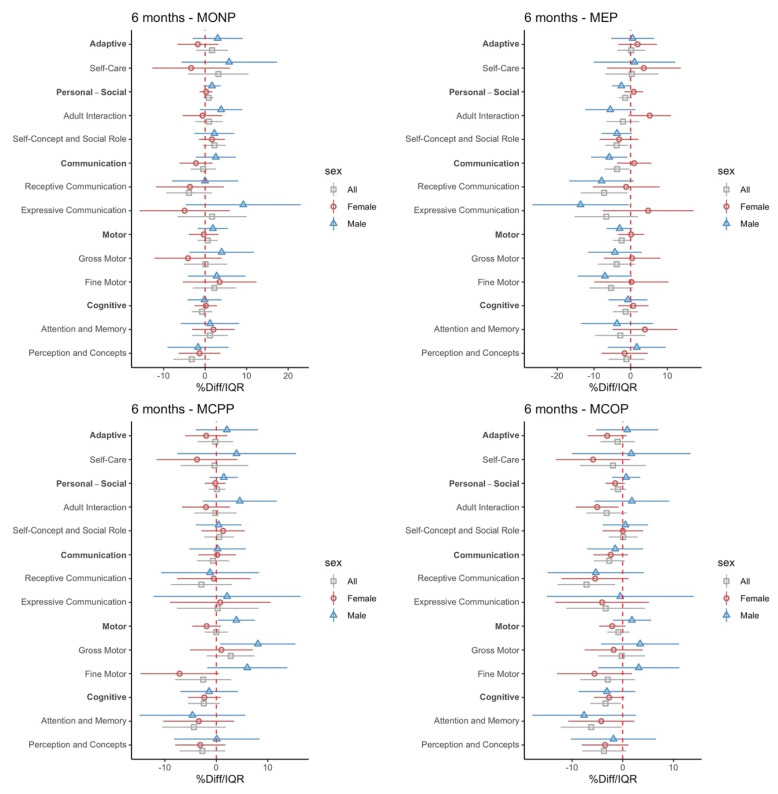

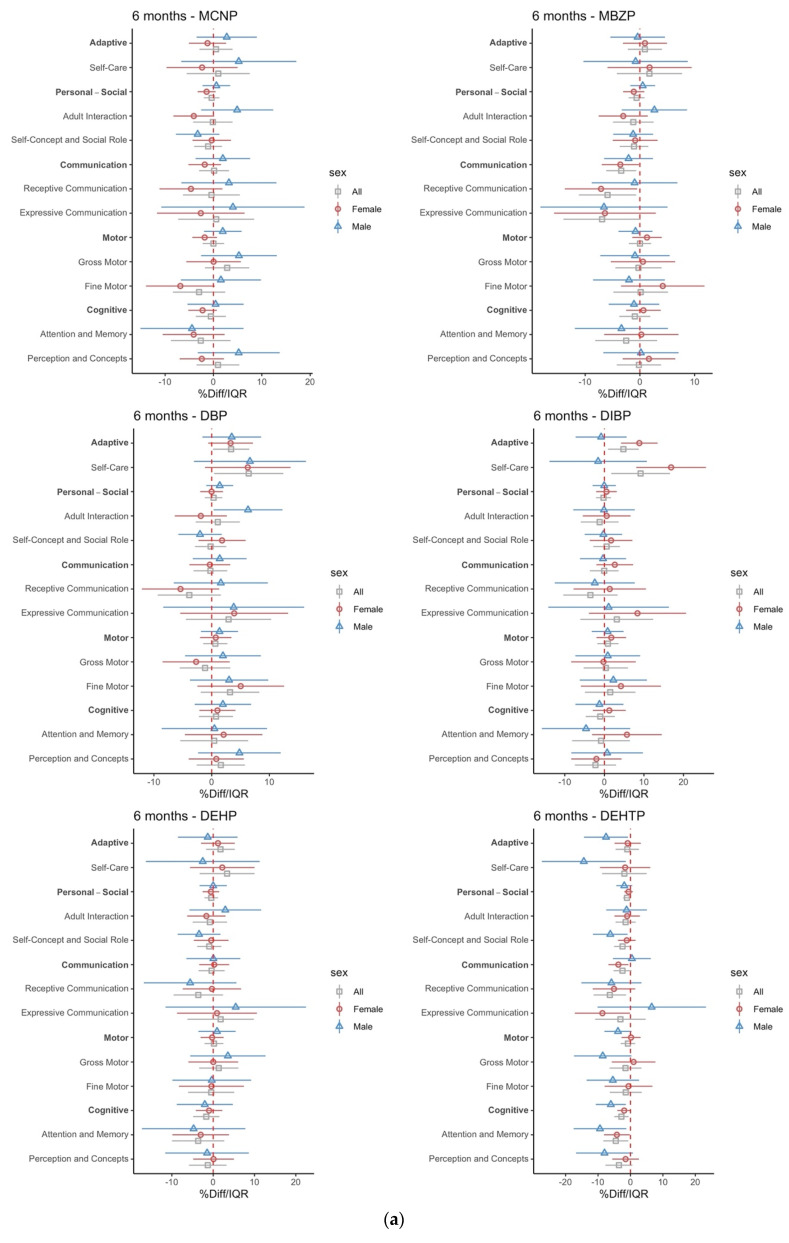

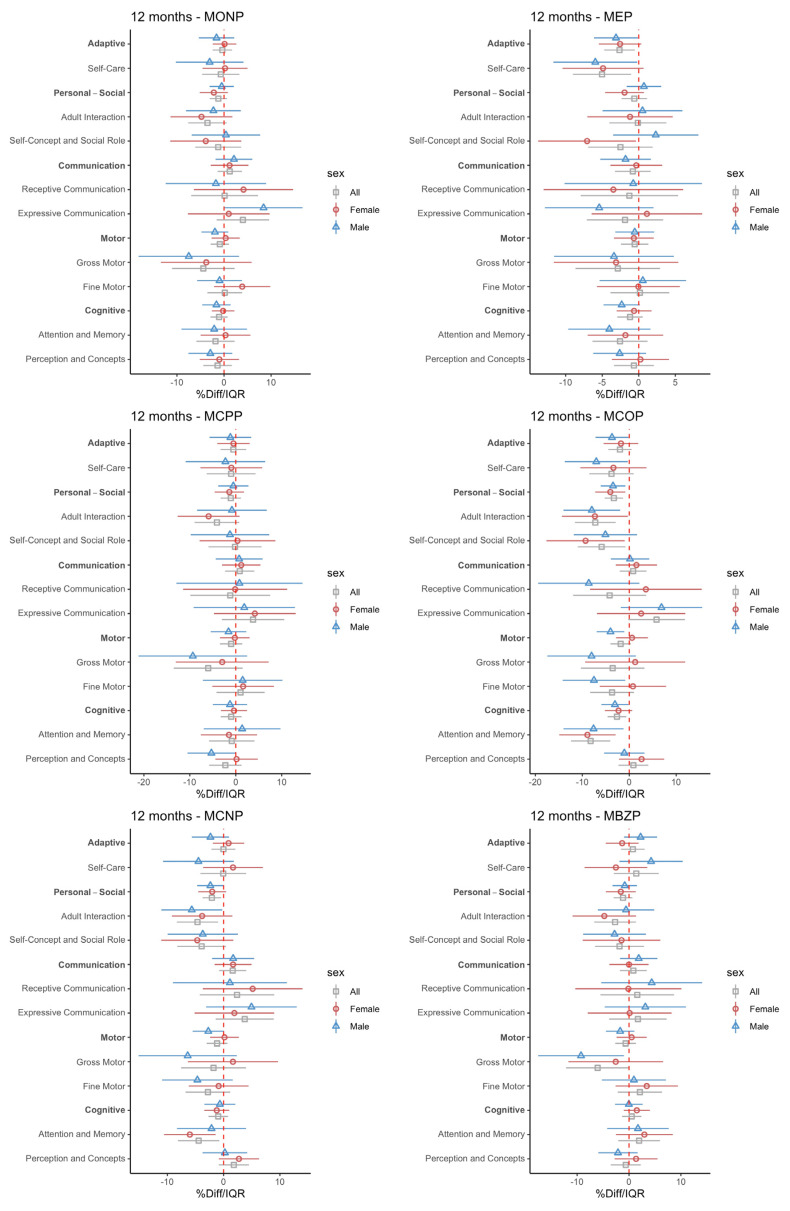

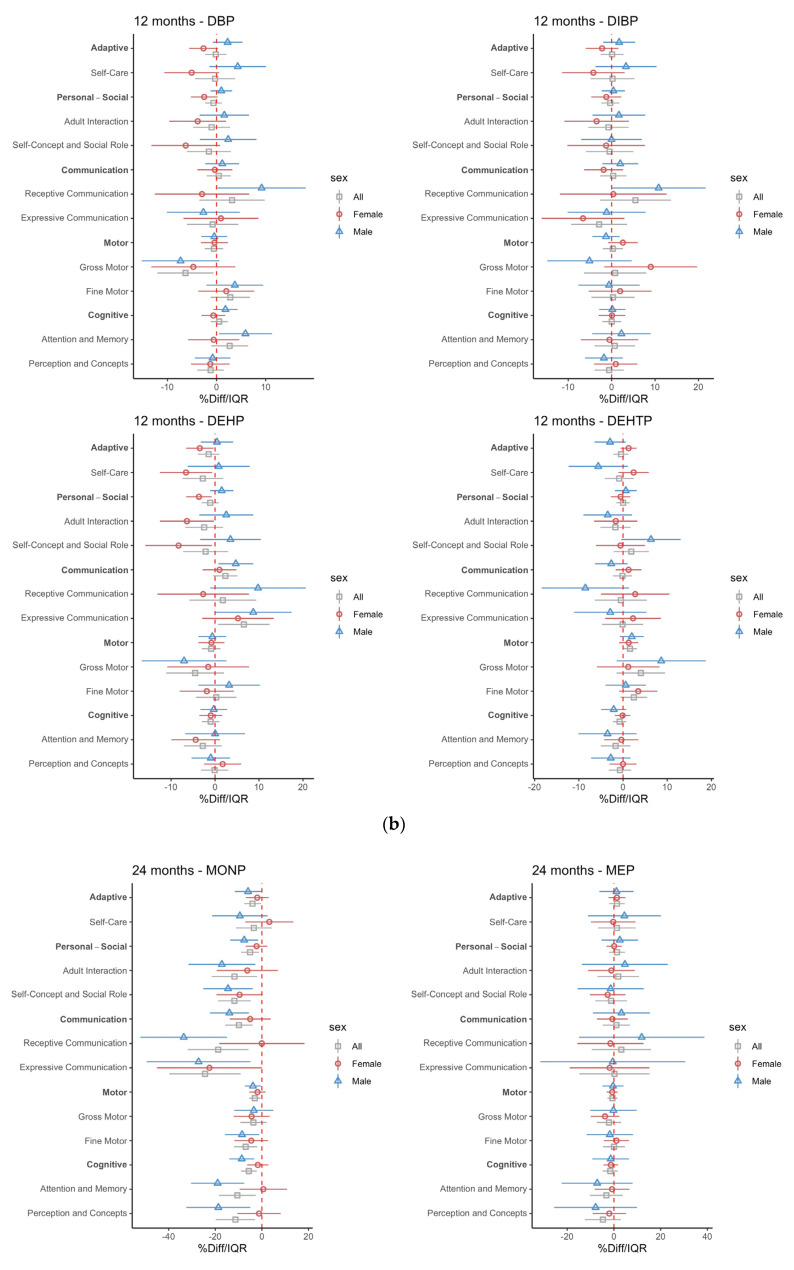

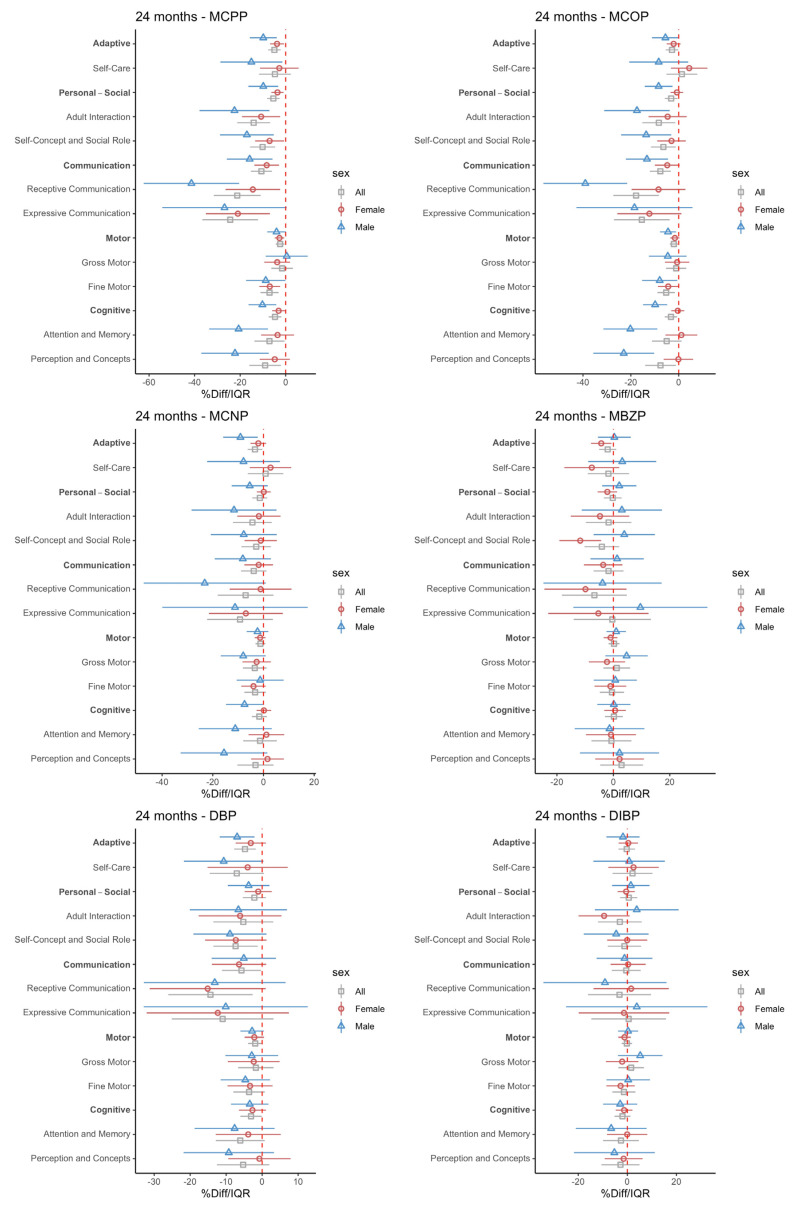

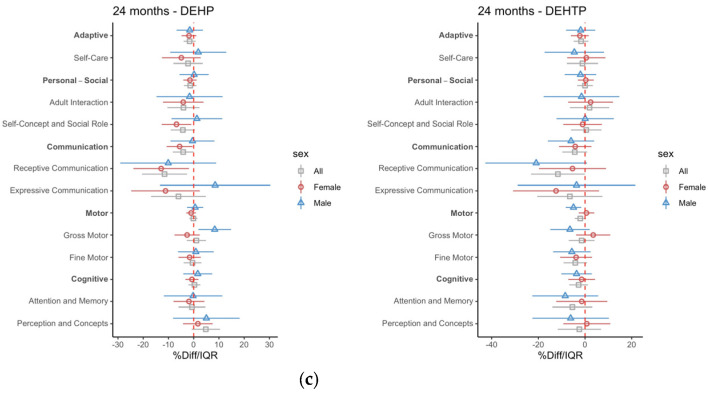
(**a**). Percentage change in BDI-2 scores at 6 months of age (n = 98) per IQR increase in the geometric mean of maternal urinary phthalate metabolite concentrations (ng/mL) across pregnancy. (**b**). Percentage change in BDI-2 scores at 12 months of age (n = 98) per IQR increase in the geometric mean of maternal urinary phthalate metabolite concentrations (ng/mL) across pregnancy. (**c**). Percentage change in BDI-2 scores at 24 months of age (n = 98) per IQR increase in the geometric mean of maternal urinary phthalate metabolite concentrations (ng/mL) across pregnancy.

Child neurodevelopment across five domains—adaptive, personal and social, communication, motor, and cognitive—were associated with various phthalate metabolites at different ages (6 months, 12 months, or 24 months), adjusting for maternal age, maternal education, and child age at assessment (Figure 3a: 6 months, Figure 3b: 12 months, Figure 3c: 24 months). For instance, an IQR increase in an in utero urinary MCOP concentration was associated with a 3.28% lower personal-social domain score at 12 months (95% CI: −5.25, −1.3), which indicates poorer performance. Interestingly, MCPP was associated with poorer performance in all five domains of BDI-2 at age 24 months. Specifically, in utero MCPP concentration was associated with 4.95% lower adaptive scores (95% CI: −7.7, −2.2), 4.69% lower cognitive (95% CI: −7.49, −1.9), 10.67% lower communication (95% CI: −15.28, −6.06), 2.45% lower motor (95% CI: −4.35, −0.55), and 5.42% lower personal-social (95% CI: −8.15, −2.69) scores per IQR increase at age 24 months, indicating holistic developmental delay in relation to this urinary metabolite.

We examined interactions between phthalate exposure and sex using cross-product terms and sex-stratified models. Sex-specificity was observed for all time points (p-interaction < 0.2) in all five domains with several phthalate metabolites. Specifically, seven among ten phthalate metabolites—MBZP, MCOP, MCNP, MCPP, MEP, ΣDIBP, ΣDEHP, ΣDEHTP—in the analysis repeatedly showed significant interaction terms throughout different time points. Stratification by infant sex revealed a distinct pattern, where a significant association was often found in one sex but not the other, leading to null associations in the combined analyses.

We found that adaptive and cognitive domains were especially sensitive to the sex-specific effects, generally showing stronger associations among boys compared to girls. For instance, metabolites of a recent phthalate replacement, ΣDEHTP, showed significant associations among boys but not girls for the adaptive domain (boys −7.53%/IQR, 95% CI: −14.58, −0.48 vs. girls −0.85%/IQR, 95% CI: −5.08, 3.37) and cognitive domain (boys -6.05%/IQR, 95% CI: −10.88, −1.22 vs. girls −1.93%/IQR, 95% CI: −4.14, 0.28) at age 6 months. In addition, at age 12 months, lower adaptive domain scores among boys were associated with MEP and MCOP, while MCOP was also associated with lower cognitive domain scores. Similarly, at age 24 months, we observed an association between ΣDBP and adaptive scores among boys but not girls. Interestingly, MONP and MCOP were associated with lower scores in all five domains at 24 months, with associations again more pronounced among boys.

We observed similar sex-specific associations in other domains as well. At 6 months of age, MEP was associated with communication domain scores (−5.84%/IQR, 95% CI: −10.93, −0.75) among boys, but not girls (0.98%/IQR, 95% CI: −3.82, 5.77). In addition, sex-stratified analysis revealed that for boys, motor scores at age 12 months were negatively associated with MCOP (boys 3.99%/IQR, 95% CI: −7, −0.99 vs. girls 0.59%/IQR, 95% CI: −2.87, 4.06) and at age 24 months with DEHTP (boys 4.95%/IQR, 95% CI: −8.29, −1.61 vs. girls 0.61%/IQR, 95% CI: −2.72, 3.94), but null associations were observed for girls.

In contrast, there are some associations where girls showed stronger relationships. MBZP, specifically, was associated with communication domain scores at 6 months (boys −2.08%/IQR, 95% CI: −6.67, 2.52 vs. girls −3.58%/IQR, 95% CI: −7.07, −0.08) and adaptive scores at age 24 months (boys 0.34%/IQR, 95% CI: −5.48, 6.16 vs. girls −4.3%/IQR, 95% CI: −7.92, −0.68) but associations were only significant among girls. It is also notable that the phthalate replacement, DEHTP, was significantly associated with communication scores at 6 months only among girls (boys 0.45%/IQR, 95% CI: −5.64, 6.53 vs. girls −3.72%/IQR, 95% CI: −6.92, −0.52), while other domain scores at the same age showed stronger associations in boys as described previously. More detailed effect estimates are shown in Supplementary Tables S4a–c.

## 4. Discussion

In this study, maternal urinary phthalate levels during pregnancy exhibited significant associations with lower BDI scores, a test of child neurodevelopment, at ages 6–24 months across five developmental domains—adaptive, personal-social, communication, motor, and cognitive. Our findings suggest that gestational exposure to phthalates and a recent phthalate replacement could negatively affect early child neurodevelopment with differences by sex. Although associations varied according to developmental age, there were some consistent relationships with specific phthalate metabolites and all five domains. Specifically, MONP and MCOP were associated with lower scores in all five domains at 24 months among boys, indicating holistic delay and sex-specificity. Similarly, MCPP was also negatively associated with all five domains at age 24 months among both sexes. As MCPP is a metabolite of several high-molecular-weight phthalates, these results indicate a possible synergetic or cumulative effect of multiple phthalates on early neurodevelopment.

The prenatal urinary concentrations of phthalate metabolites among PROTECT study participants have been reported and discussed in previous studies [30]. Average BDI domain DQ scores and subdomain scaled scores in the analysis group are within the normal range (within one standard deviation of mean: 100 ± 15 for domain DQ scores and 10 ± 3 for subdomain scaled scores) for all age points, although we observed slightly lower scores of cognitive domain and subdomain at age 24 months compared to other age points (cognitive domain: 86.41 ± 9.99; attention and memory subdomain: 7.42 ± 1.93; perception and concepts subdomain: 7.57 ± 2.12). Cognitive domain and subdomain scores at 6 months and 12 months showed similar distributions.

While studies that used BDI as a neurodevelopmental assessment are limited, previous studies of in utero phthalate exposure and child neurodevelopment reported statistically significant associations between several phthalate metabolites and neurodevelopmental delay in cognitive, motor, and peer relationships in early to mid-childhood [39,40,41,42]. In addition, statistically significant sex-specificity has been reported frequently in previous studies. A study in South Korea found that DEHP metabolites were negatively associated with a mental index of boys at age 6 months [43], and a study of children in Mexico City found that metabolites of DEHP were associated with poorer motor and cognitive scale scores at 48 months of age, with stronger associations observed among boys compared to girls [44]. A study of French mother–son pairs found that MBP was associated with higher internalizing behaviors, relationship problems, and emotional symptoms scores at age 3 years [45]. On the other hand, a few studies suggested stronger associations among girls. Another study of children in Mexico showed third trimester exposure to DEHP was significantly associated with lower mental index scores among girls at 36 months [46], and the Mount Sinai Children’s Environmental Health Study showed that MCPP and MBZP were associated with mental and psychomotor indices at age 24 months specifically in girls [47]. Interestingly, an inconsistency exists for DBP metabolites as significant associations in girls were observed in the Mount Sinai Children’s Environmental Health study [47], while another study from South Korea showed stronger associations among boys at 6 months [43]. In addition, Loftus et al. found no significant associations of gestational exposure to MCOP and MCPP with child language and cognitive ability at age 3 to 6 years. Overall, some inconsistencies exist in previous results, especially in the sex-specific effects of phthalate exposure on child neurodevelopment [48]. The differences may come from differences in the age at neurodevelopmental assessment within each study or differences in phthalate exposure at the population level. Further research is needed pertaining to the role of sex in the relationships between gestational phthalate exposure and child neurodevelopment.

There are a number of potential mechanisms that underlie the associations observed in this study. Phthalates may disrupt the maternal thyroid signaling pathway [49,50,51], which is crucial for early brain development [52]; thus, abnormal maternal thyroid function is associated with child neurodevelopmental delay. Phthalate exposure is also associated with altered sex hormone levels during gestation, which can affect child neurodevelopment by damaging neurotransmitters [6], disrupting development of sex-specific brain structures [53,54], or altering the medial prefrontal cortex function [55,56]. In addition, phthalate exposure may affect child neurodevelopment by causing oxidative stress. Prenatal phthalate exposure has been associated with increased oxidative stress during pregnancy [57], and animal studies have suggested that increased maternal oxidative stress during pregnancy can alter offspring brain development [58,59]. In addition, animal studies have indicated that phthalates can alter the lipid metabolism of the fetal brain [60]. Gestational phthalate exposure has been related to increased lipid peroxidation during pregnancy [57,61], which can lead to motor neuron death and brain disorders [62]. While epidemiological studies of the health effects of phthalate replacements are extremely limited, recent in vitro studies have demonstrated that DEHTP may increase oxidative stress and inflammatory response [63].

A strength of our study is the extensive panel of phthalate and phthalate replacement metabolites, given the limited research on the effects of human exposure to DEHTP. In addition, few if any studies have used the BDI-2 to assess potential changes in neurodevelopment due to gestational phthalate exposure, which enabled us to examine a broad spectrum of child neurodevelopmental outcomes, including adaptive, personal-social, communication, motor, and cognitive domains. Furthermore, we assessed exposures in relation to neurodevelopment early in infancy, which few previous studies have reported. Lastly, our study utilized the PROTECT birth cohort in Puerto Rico, where the population has a high burden of environmental contamination, poverty, and adverse health outcomes, and historically has been medically underserved. Our results will support research translation efforts and inform the development of actionable preventive measures within this vulnerable population.

A major limitation of our study is the small sample size. First, we were not able to evaluate critical windows of susceptibility to exposure across gestation or non-linear relationships, but future work with a larger sample will improve our ability to explore these relationships in more detail. Similarly, we had a relatively small number of individuals with repeated assessments at multiple time points, and we were therefore not able to assess changes over time. Although we observed varying associations between phthalate metabolites and BDI-2 scores at different time points, it is unclear whether this discrepancy came from the inability to detect the relationships at earlier time points due to insufficient sample sizes, or the progression of adverse effects over time. In addition, there were some differences in demographic characteristics between participants with assessments at the different time points, which may at least partially explain our results. However, all models were adjusted for maternal age and education. Therefore, a longitudinal study with a single analysis group to assess the impact of gestational phthalate exposure on neurodevelopmental trajectories is needed. Moreover, our analysis was limited to single pollutants due to the small sample size; however, chemical exposures occur as mixtures and certain phthalates share similar exposure patterns, calling for a need to study cumulative effects. Lastly, even though we observed a trend of stronger associations in boys, the sex-specificity was not conclusive. Thus, further study using advanced mixture methods and elucidating biological mechanisms of sex-dependency will enhance our findings.

## 5. Conclusions

In conclusion, our results provide evidence supporting the hypothesis that maternal exposure to phthalates or phthalate replacements in pregnancy may have adverse effects on child neurodevelopment across multiple domains during infancy. In addition, our results indicate that these associations may vary by child age and sex. Our findings align with previous studies reporting associations between prenatal phthalate exposure and neurodevelopmental delay in childhood and its dimorphism by sex. This highlights the need for both preventive measures against environmental exposures as well as for accessible, early interventions in developmental delays in Puerto Rico and other marginalized communities. Further research pertaining to the long-term effects of in utero exposure to phthalates on neurodevelopment over early childhood, and elucidating biological underlying mechanisms is essential.

## Figures and Tables

**Table 1:** Demographic characteristics of study participants from the Puerto Rico PROTECT cohort (N = 1576) and analysis group (N = 274).

		PROTECT (N = 1576)	Analysis Group (N = 274)
Maternal Characteristics		n (%)	n (%)
Alcohol Use	Never	836 (53)	125 (46)
	Pre-pregnancy	608 (39)	123 (45)
Currently Employed	No	573 (36)	75 (27)
	Yes	953 (60)	185 (68)
Maternal Education	GED or less	337 (21)	29 (11)
	Some college	515 (33)	98 (36)
	Bachelors or higher	674 (43)	130 (47)
Annual Income	<10 k	431 (27)	52 (19)
	10 k– < 30 k	413 (26)	84 (31)
	30 k– < 50 k	300 (19)	63 (23)
	≥50 k	195 (12)	34 (12)
Maternal Age (years)	18–24	583 (37)	68 (25)
	25–29	490 (31)	91 (33)
	30–34	324 (21)	58 (21)
	35–41	173 (11)	43 (16)
Marital Status	Single	302 (19)	27 (10)
	Married	805 (51)	151 (55)
	Cohabitating	422 (27)	78 (28)
BMI	≤25	755 (48)	128 (47)
	>25 to <30	427 (27)	76 (28)
	≥30	289 (18)	43 (16)
Number of Children	0	651 (41)	109 (40)
	1	534 (34)	79 (29)
	2–5	347 (22)	72 (26)
Child Characteristics		Mean ± SD or n (%)	
Gestational Age (weeks)		38.9 ± 1.9	39.0 ± 1.7
Birth Weight (grams)		3174 ± 547	3162.47 ± 564
Sex	Female	521 (33)	137 (50)
	Male	566 (36)	137 (50)

## Data Availability

The data that were used in this study can be made accessible to researchers upon appropriate request with restrictions to ensure the privacy of human subjects.

## Supplementary Materials

The following supporting information can be downloaded at: https://www.mdpi.com/article/10.3390/toxics11010065/s1, Figure S1. Distribution of prenatal phthalate concentration averaged over gestation by age group; Figure S2. Spaghetti plots of the change of BDI domain/subdomain score of children whose score were reported twice (at age around 12 months and 24 months) during the study period. Brown solid lines indicate the change in median score. Table S1. Demographic characteristics of study sample (N = 274); Table S2. Distributions of BDI scores among analysis group (n = 274). Arithmetic means and standard deviation by age group; Table S3. Comparison of uncorrected urinary phthalate and phthalate replacement metabolite concentrations (ng/mL) in study sample (N = 274) by study visit; Table S4. a: Percentage change in BDI-2 scores per IQR increase in the geometric mean of maternal urinary phthalate metabolite concentrations (ng/mL) across pregnancy from 6 months group (n all = 98, n girls = 49, n boys = 49); Table S4. b: Percentage change in BDI-2 scores per IQR increase in the geometric mean of maternal urinary phthalate metabolite concentrations (ng/mL) across pregnancy from 12 months group (n all = 130, n girls = 64, n boys = 66); Table S4. c: Percentage change in BDI-2 scores per IQR increase in the geometric mean of maternal urinary phthalate metabolite concentrations (ng/mL) across pregnancy from 24 months group (n all = 123, n girls = 69, n boys = 54).
