# Supplementary material for: Gestational Exposure to Phthalates and Phthalate Replacements in Relation to Neurodevelopmental Delays in Early Childhood
Source: Toxics. 2023 Jan 11;11(1):65. doi: 10.3390/toxics11010065 (PMC9863718; doi:10.3390/toxics11010065)
Supplement: Supplementary file 1 [file toxics-11-00065-s001.zip › toxics-2022719-SI.pdf]

Supplementary Materials

Title

Gestational exposure to phthalates and phthalate replacements in relation to neurodevelopmental delays in early childhood

Authors

Seonyoung Park, Emily Zimmerman, Gredia Huerta-Montañez, Zaira Rosario Pabon, Carmen M. Vélez-Vega, José F. Cordero, Akram Alshawabkeh, John D. Meeker, Deborah J. Watkins

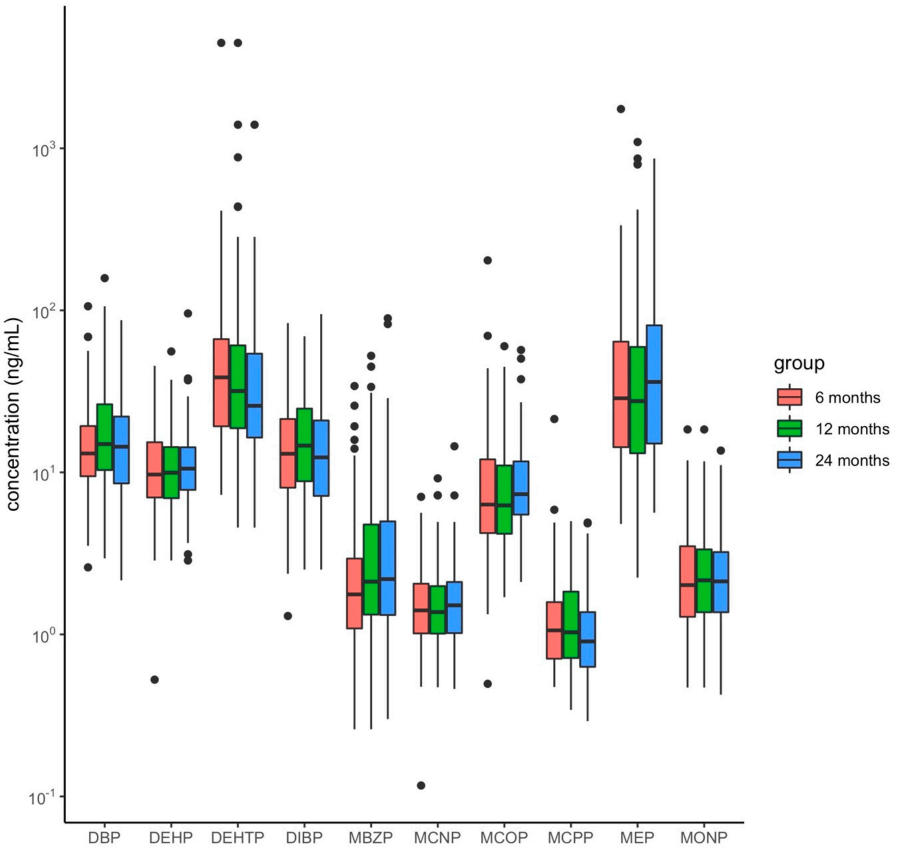

Figure S1. Distribution of prenatal phthalate concentration averaged over gestation by age group.

Table S1. Demographic characteristics of study sample (N=274).

		**6 months**			**12 months**			24 months		
		**All (n=98)**	**Female (n=49)**	**Male (n=49)**	**All (n=130)**	**Female (n=64)**	**Male (n=66)**	**All (n=123)**	**Female (n=69)**	**Male (n=54)**
Population Characteristics		n (%)								
Alcohol Use	Never	43 (43.9)	22 (44.9)	21 (42.9)	68 (52.3)	36 (56.2)	32 (48.5)	54 (43.9)	28 (40.6)	26 (48.1)
	Yes, before pregnancy	49 (50.0)	24 (49.0)	25 (51.0)	56 (43.1)	27 (42.2)	29 (43.9)	52 (42.3)	31 (44.9)	21 (38.9)
Currently Employed	No	25 (25.5)	8 (16.3)	17 (34.7)	38 (29.2)	16 (25.0)	22 (33.3)	33 (26.8)	17 (24.6)	16 (29.6)
	Yes	73 (74.5)	41 (83.7)	32 (65.3)	92 (70.8)	48 (75.0)	44 (66.7)	76 (61.8)	44 (63.8)	32 (59.3)
Maternal Education	GED or less	11 (11.2)	4 (8.2)	7 (14.3)	19 (14.6)	11 (17.2)	8 (12.1)	14 (11.4)	7 (10.1)	7 (13.0)
	Some college	30 (30.6)	15 (30.6)	15 (30.6)	48 (36.9)	22 (34.4)	26 (39.4)	43 (35.0)	25 (36.2)	18 (33.3)
	Bachelors or higher	56 (57.1)	29 (59.2)	27 (55.1)	62 (47.7)	30 (46.9)	32 (48.5)	51 (41.5)	29 (42.0)	22 (40.7)
Annual Household Income	<10k	16 (16.3)	6 (12.2)	10 (20.4)	30 (23.1)	14 (21.9)	16 (24.2)	25 (20.3)	13 (18.8)	12 (22.2)
	10k - <30k	31 (31.6)	16 (32.7)	15 (30.6)	40 (30.8)	22 (34.4)	18 (27.3)	35 (28.5)	22 (31.9)	13 (24.1)
	30k - <50k	29 (29.6)	15 (30.6)	14 (28.6)	33 (25.4)	15 (23.4)	18 (27.3)	23 (18.7)	11 (15.9)	12 (22.2)
	≥50k	14 (14.3)	9 (18.4)	5 (10.2)	14 (10.8)	9 (14.1)	5 (4.6)	13 (10.6)	9 (13.0)	4 (7.4)
Maternal Age (years)	18-24	21 (21.4)	12 (24.5)	9 (18.4)	36 (27.7)	15 (23.4)	21 (31.8)	31 (25.2)	17 (24.6)	14 (25.9)
	25-29	33 (33.7)	14 (28.6)	19 (38.8)	44 (33.8)	24 (37.5)	20 (30.3)	39 (31.7)	24 (34.8)	15 (27.8)
	30-34	25 (25.5)	13 (26.5)	12 (24.5)	29 (22.3)	13 (20.3)	16 (24.2)	23 (18.7)	11 (15.9)	12 (22.2)
	35-41	19 (19.4)	10 (20.4)	9 (18.4)	21 (16.2)	12 (18.8)	9 (13.6)	16 (13.0)	9 (13.0)	7 (13.0)
Marital Status	Single	8 (8.2)	4 (8.2)	4 (8.2)	10 (7.7)	5 (7.8)	5 (7.6)	14 (11.4)	8 (11.6)	6 (11.1)
	Married	64 (65.3)	32 (65.3)	32 (65.3)	72 (55.4)	33 (51.6)	39 (59.1)	66 (53.7)	35 (50.7)	31 (57.4)
	Cohabitating	24 (24.5)	12 (24.5)	12 (24.5)	46 (35.4)	25 (39.1)	21 (31.8)	28 (22.8)	17 (24.6)	11 (20.4)
BMI	≤ 25	45 (45.9)	17 (34.7)	28 (57.1)	65 (50.0)	30 (46.9)	35 (53.0)	53 (43.1)	28 (40.6)	25 (46.3)
	> 25 to < 30	31 (31.6)	20 (40.8)	11 (22.4)	34 (26.2)	16 (25.0)	18 (27.3)	33 (26.8)	23 (33.3)	10 (18.5)
	≥ 30	20 (20.4)	11 (22.4)	9 (18.4)	22 (16.9)	15 (23.4)	7 (10.6)	19 (15.4)	8 (11.6)	11 (20.4)
Number of Children	0	36 (36.7)	19 (38.8)	17 (34.7)	62 (47.7)	28 (43.7)	34 (51.5)	39 (31.7)	27 (39.1)	12 (22.2)
	1	37 (37.8)	19 (38.8)	18 (36.7)	41 (31.5)	22 (34.4)	19 (28.8)	33 (26.8)	16 (23.2)	17 (31.5)
	2-5	25 (25.5)	11 (22.4)	14 (28.6)	27 (20.8)	14 (21.9)	13 (19.7)	37 (30.1)	18 (26.1)	19 (35.2)

Table S2. Distributions of BDI scores among analysis group (n=274). Arithmetic means and standard deviation by age group.

Domain / Subdomain	67 months (n=98)	12 months (n=130)	24 months (n=123)
**Adaptive Domain**	102.53 ± 14.98	102.12 ± 10.23	97.28 ± 11.71
Self-Care	10.51 ± 3	10.42 ± 2.05	8.48 ± 2.2
**Personal-Social Domain**	108.65 ± 7.17	105.61 ± 8.93	101.86 ± 12.31
Adult Interaction	12.4 ± 2.15	11.38 ± 2	10.61 ± 3.35
Self-Concept and Social Role	10.97 ± 1.29	10.77 ± 2.19	10.68 ± 2.49
**Communication Domain**	97.04 ± 11.76	99.06 ± 11.66	90.34 ± 18.67
Receptive Communication	8.78 ± 2.1	8.57 ± 2.56	7.86 ± 3.92
Expressive Communication	10.13 ± 3.1	11.09 ± 2.67	8.37 ± 3.99
**Motor Domain**	105.49 ± 9.77	103.65 ± 9.14	102.56 ± 8.21
Fine Motor	10.59 ± 2.39	11.28 ± 2.27	11.16 ± 2.07
Gross Motor	11.48 ± 2.3	10.09 ± 2.4	9.87 ± 1.78
**Cognitive Domain**	100.06 ± 12.18	100.79 ± 8.95	86.41 ± 9.99
Attention and Memory	9.61 ± 2.44	10.33 ± 2	7.42 ± 1.93
Perception and Concepts	10.41 ± 1.94	9.92 ± 1.34	7.57 ± 2.12

Table S3. Comparison of uncorrected urinary phthalate and phthalate replacement metabolite concentrations (ng/ml) in study sample (N=274) by study visit. One-way ANOVA tests were used to test for differences in means between visits

phthalate	visit	N	n<LOD	%<LOD	GM	GSD	25th	50th	75th	p-value
MBP	Total	645	3	0	12.53	3.09	5.9	13.8	26.3	0.976
MBP	1	228	0	0	13.04	2.91	5.8	13.35	26.22	
MBP	2	231	1	0	12.27	3.11	5.35	13.9	26.5	
MBP	3	186	2	1	12.24	3.29	7	14.65	26.2	
MBZP	Total	645	36	6	2.33	3.83	1	2.1	5.1	0.864
MBZP	1	228	9	4	2.37	3.72	1	2.15	5.1	
MBZP	2	231	13	6	2.27	3.87	1	2.2	5	
MBZP	3	186	14	8	2.35	3.95	1	2	4.8	
MCNP	Total	645	13	2	1.34	2.51	0.8	1.3	2.2	0.655
MCNP	1	228	1	0	1.46	2.39	0.9	1.4	2.3	
MCNP	2	231	8	3	1.22	2.57	0.7	1.2	2	
MCNP	3	186	4	2	1.35	2.57	0.8	1.35	2.28	
MCOP	Total	645	0	0	7.02	3.23	3.2	6	13.4	0.495
MCOP	1	228	0	0	8.05	3.05	3.82	6.6	16.42	
MCOP	2	231	0	0	6.38	3.4	2.7	5.7	12	
MCOP	3	186	0	0	6.69	3.24	3.23	5.6	12.05	
MCPP	Total	645	135	21	1.01	2.66	0.5	1	1.8	0.468
MCPP	1	228	39	17	1.09	2.66	0.6	1	1.83	
MCPP	2	231	51	22	0.95	2.62	0.5	0.9	1.8	
MCPP	3	186	45	24	0.99	2.71	0.43	1	1.9	
MECPP	Total	645	1	0	10.48	2.5	6	10.5	18.2	0.564
MECPP	1	228	0	0	11.12	2.4	6.5	10.5	18.68	
MECPP	2	231	1	0	10	2.48	6	10.1	17.65	
MECPP	3	186	0	0	10.32	2.65	5.5	10.9	18.08	
MECPTP	Total	497	0	0	28.76	3.98	11.3	22.1	57.6	0.571
MECPTP	1	174	0	0	29.05	4.23	10.2	21.95	56.42	
MECPTP	2	176	0	0	28.49	3.89	12.3	22.9	55.88	
MECPTP	3	147	0	0	28.75	3.84	11.55	22.5	59.15	
MEHHP	Total	645	5	1	5.72	2.79	3.2	5.9	10.5	0.97
MEHHP	1	228	1	0	6.03	2.7	3.4	6.2	10.6	
MEHHP	2	231	2	1	5.52	2.79	3.1	5.8	10.3	
MEHHP	3	186	2	1	5.62	2.92	3.2	6.15	10.45	
MEHHTP	Total	497	8	2	4.8	4.05	1.8	4.2	10.6	0.372
MEHHTP	1	174	2	1	5.17	4.14	1.72	4.25	10.35	
MEHHTP	2	176	4	2	4.69	4.02	1.78	4.65	10.53	
MEHHTP	3	147	2	1	4.53	4	1.8	3.7	10.9	
MEHP	Total	645	140	22	1.94	2.6	0.9	1.9	3.6	0.537
MEHP	1	228	49	21	1.95	2.53	0.98	1.85	3.62	
MEHP	2	231	48	21	1.89	2.57	0.9	1.9	3.3	
MEHP	3	186	43	23	1.99	2.73	0.9	1.9	3.7	

MEOHP	Total	645	1	0	5.17	2.79	3	5.6	9.7	0.84
MEOHP	1	228	0	0	5.23	2.69	2.9	5.55	8.93	
MEOHP	2	231	1	0	5.02	2.8	2.95	5.2	9.95	
MEOHP	3	186	0	0	5.28	2.9	3.12	5.9	9.78	
MEP	Total	645	1	0	31.14	4.83	9.7	23.7	79	0.881
MEP	1	228	0	0	32.05	4.43	9.85	23.55	79.1	
MEP	2	231	0	0	30.13	4.84	9.05	23.7	77.7	
MEP	3	186	1	1	31.33	5.36	10.2	25.5	78.18	
MHBP	Total	640	107	17	1.32	2.93	0.6	1.3	2.8	0.952
MHBP	1	227	31	14	1.38	2.88	0.6	1.4	2.95	
MHBP	2	228	40	18	1.26	2.94	0.5	1.3	2.62	
MHBP	3	185	36	19	1.34	2.99	0.6	1.4	2.8	
MHIBP	Total	640	12	2	3.64	2.81	1.8	3.7	7.73	0.984
MHIBP	1	227	3	1	3.66	2.78	1.7	3.6	8.5	
MHIBP	2	228	3	1	3.59	2.81	1.87	3.6	7.23	
MHIBP	3	185	6	3	3.67	2.86	2	4	7.5	
MIBP	Total	645	11	2	8.46	2.89	4.1	9	18.3	0.746
MIBP	1	228	1	0	8.34	2.85	4	8.9	19	
MIBP	2	231	4	2	8.45	2.94	4.05	8.5	18.2	
MIBP	3	186	6	3	8.61	2.9	4.3	9.8	16.8	
MONP	Total	497	32	6	2.04	3.35	1	1.8	4	0.924
MONP	1	174	7	4	2.07	3.14	0.92	1.7	3.95	
MONP	2	176	12	7	1.9	3.37	0.8	1.7	4.2	
MONP	3	147	13	9	2.17	3.58	1.1	1.9	3.85	

Table S4. a: Percent change in BDI-2 scores at 6 months per IQR increase in the geometric mean of maternal urinary phthalate metabolite concentrations (ng/ml) across pregnancy (n all = 98, n girls=49, n boys=49).

Domain/Subdomain	sex	MBZP	MCOP	MONP	MCNP	MCPP	MEP	DEHP	DBP	DIBP	DEHTP
**Adaptive Domain**	All	0.91 (-2.25,4.08)	-1.01 (-4.45,2.43)	1.66 (-2.22,5.55)	0.52 (-2.94,3.98)	-0.18 (-3.67,3.31)	0.17 (-3.68,4.01)	1.76 (-1.76,5.29)	3.36 (0.18,6.55)	4.8 (0.86,8.74)	-0.98 (-4.64,2.69)
**Adaptive Domain**	Female	0.93 (-3.22,5.08)	-3.07 (-7.03,0.89)	-1.77 (-6.88,3.34)	-1.23 (-5.2,2.74)	-1.96 (-6.19,2.26)	1.88 (-3.53,7.3)	1.15 (-3.07,5.36)	3.27 (-0.72,7.27)	8.83 (4.09,13.58)	-0.85 (-5.08,3.37)
**Adaptive Domain**	Male	-0.41 (-5.55,4.73)	0.89 (-5.41,7.18)	3.03 (-3.25,9.3)	2.74 (-3.67,9.16)	2.06 (-4.16,8.28)	0.53 (-5.45,6.52)	-1.32 (-8.75,6.11)	3.47 (-1.76,8.7)	-0.83 (-7.47,5.81)	-7.53 (-14.58,-0.48)
Self-Care	All	1.74 (-4.3,7.79)	-1.93 (-8.49,4.63)	3.18 (-4.24,10.59)	1 (-5.61,7.6)	-0.34 (-7,6.32)	0.31 (-7.03,7.67)	3.36 (-3.37,10.1)	6.41 (0.34,12.49)	9.16 (1.64,16.68)	-1.87 (-8.86,5.12)
Self-Care	Female	1.77 (-6.14,9.69)	-5.85 (-13.41,1.71)	-3.38 (-13.13,6.38)	-2.35 (-9.92,5.22)	-3.75 (-11.82,4.32)	3.6 (-6.74,13.92)	2.19 (-5.85,10.24)	6.25 (-1.38,13.87)	16.86 (7.81,25.93)	-1.63 (-9.7,6.43)
Self-Care	Male	-0.78 (-10.6,9.02)	1.69 (-10.33,13.71)	5.77 (-6.21,17.76)	5.23 (-7.01,17.49)	3.94 (-7.94,15.81)	1.02 (-10.41,12.45)	-2.52 (-16.7,11.67)	6.63 (-3.37,16.62)	-1.59 (-14.26,11.09)	-14.37 (-27.83,-0.91)
**Personal-Social Domain**	All	-0.61 (-2.09,0.88)	-0.92 (-2.53,0.69)	0.82 (-0.42,2.07)	-0.38 (-2.02,1.26)	0.13 (-1.52,1.77)	-1.5 (-3.28,0.28)	-0.47 (-2.14,1.2)	0.33 (-1.21,1.87)	-0.23 (-2.14,1.69)	-1.08 (-2.27,0.11)
**Personal-Social Domain**	Female	-1.12 (-3.12,0.87)	-1.5 (-3.42,0.43)	0.24 (-1.37,1.85)	-1.42 (-3.33,0.49)	-0.2 (-2.29,1.89)	0.85 (-1.79,3.49)	-0.56 (-2.62,1.51)	-0.01 (-2.05,2.02)	0.52 (-2.15,3.19)	-0.6 (-1.98,0.77)
**Personal-Social Domain**	Male	0.52 (-1.83,2.86)	0.68 (-2.19,3.54)	1.65 (-0.5,3.8)	0.59 (-2.36,3.54)	1.46 (-1.36,4.28)	-2.55 (-5.16,0.07)	0 (-3.4,3.39)	1.39 (-1.01,3.79)	-0.05 (-3.08,2.98)	-1.91 (-4.46,0.65)
Adult Interaction	All	-1.21 (-4.98,2.56)	-3.18 (-7.23,0.87)	0.93 (-2.44,4.32)	-0.13 (-4.26,3.99)	-0.17 (-4.33,3.99)	-2.09 (-6.64,2.48)	-0.82 (-5.04,3.4)	1.05 (-2.82,4.93)	-1.2 (-6.04,3.64)	-1.49 (-4.65,1.68)
Adult Interaction	Female	-3.05 (-7.67,1.57)	-5.05 (-9.39,-0.71)	-0.64 (-5.6,4.33)	-4.06 (-8.4,0.27)	-2.01 (-6.81,2.79)	5.19 (-0.77,11.15)	-1.65 (-6.42,3.12)	-1.88 (-6.52,2.76)	0.58 (-5.64,6.79)	-1.02 (-5.1,3.05)
Adult Interaction	Male	2.65 (-3.5,8.8)	1.82 (-5.76,9.4)	3.84 (-1.49,9.17)	4.9 (-2.77,12.56)	4.57 (-2.84,11.99)	-5.56 (-12.58,1.46)	2.93 (-6.01,11.87)	6.3 (0.16,12.45)	-0.1 (-8.11,7.92)	-1.19 (-7.72,5.35)
Self-Concept and Social Role	All	-1.07 (-3.73,1.58)	0.09 (-2.81,2.98)	2.22 (-0.58,5.03)	-1.11 (-4.03,1.8)	0.57 (-2.36,3.51)	-3.81 (-6.93,-0.69)	-0.95 (-3.93,2.02)	-0.19 (-2.94,2.56)	0.56 (-2.86,3.97)	-2.44 (-5.12,0.24)
Self-Concept and Social Role	Female	-0.87 (-5.09,3.36)	0.02 (-4.12,4.17)	1.63 (-1.62,4.89)	-0.36 (-4.46,3.74)	1.31 (-3.02,5.66)	-3.15 (-8.58,2.3)	-0.48 (-4.81,3.83)	1.8 (-2.41,6)	1.69 (-3.87,7.24)	-1.14 (-3.95,1.69)
Self-Concept and Social Role	Male	-1.24 (-4.98,2.51)	0.52 (-4.09,5.13)	2.22 (-2.77,7.22)	-3.26 (-7.89,1.37)	0.45 (-4.13,5.02)	-3.73 (-7.96,0.5)	-3.41 (-8.75,1.93)	-2.02 (-5.88,1.84)	-0.26 (-5.13,4.6)	-6.22 (-11.78,-0.65)
**Communication Domain**	All	-3.42 (-6.2,-0.64)	-2.67 (-5.73,0.4)	-0.46 (-3.52,2.6)	0.12 (-3.01,3.26)	-0.64 (-3.79,2.52)	-3.71 (-7.1,-0.32)	-0.39 (-3.6,2.82)	-0.22 (-3.17,2.73)	-0.05 (-3.73,3.63)	-2.45 (-5.27,0.37)
**Communication Domain**	Female	-3.58 (-7.07,-0.08)	-2.38 (-5.9,1.14)	-2.21 (-6.36,1.94)	-1.8 (-5.28,1.67)	0.18 (-3.59,3.95)	0.98 (-3.82,5.77)	0.24 (-3.49,3.98)	-0.32 (-3.96,3.32)	2.62 (-2.15,7.4)	-3.72 (-6.92,-0.52)
**Communication Domain**	Male	-2.08 (-6.67,2.52)	-1.48 (-7.15,4.18)	2.57 (-2.47,7.6)	1.94 (-3.87,7.75)	0.24 (-5.4,5.88)	-5.84 (-10.93,-0.75)	0.04 (-6.67,6.75)	1.39 (-3.41,6.19)	-0.34 (-6.33,5.65)	0.45 (-5.64,6.53)
Receptive Communication	All	-5.92 (-11.24,-0.62)	-7.15 (-12.88,-1.42)	-3.89 (-9.46,1.68)	-0.42 (-6.37,5.55)	-2.89 (-8.87,3.08)	-7.23 (-13.67,-0.78)	-3.61 (-9.66,2.45)	-3.88 (-9.43,1.67)	-3.53 (-10.5,3.42)	-6.36 (-11.44,-1.28)
Receptive Communication	Female	-7.1 (-13.9,-0.3)	-5.44 (-12.24,1.36)	-3.66 (-12.16,4.83)	-4.65 (-11.36,2.05)	-0.48 (-7.84,6.86)	-1.23 (-10.61,8.12)	-0.3 (-7.58,6.98)	-5.4 (-12.29,1.5)	1.36 (-8.07,10.79)	-5.08 (-11.91,1.73)
Receptive Communication	Male	-0.97 (-9.04,7.09)	-5.32 (-15.08,4.43)	-0.02 (-8.33,8.28)	3.19 (-6.92,13.31)	-1.22 (-11.03,8.6)	-7.92 (-17,1.15)	-5.57 (-17.12,5.98)	1.59 (-6.79,9.97)	-2.43 (-12.84,7.98)	-5.86 (-15.49,3.76)
Expressive Communication	All	-6.91 (-14.06,0.26)	-3.36 (-11.26,4.54)	1.67 (-6.8,10.15)	0.57 (-7.41,8.55)	0.26 (-7.78,8.3)	-6.62 (-15.37,2.13)	1.8 (-6.36,9.96)	2.93 (-4.56,10.41)	3.13 (-6.22,12.47)	-3.1 (-11.04,4.86)
Expressive Communication	Female	-6.39 (-15.97,3.18)	-4.07 (-13.57,5.44)	-4.94 (-16.23,6.36)	-2.61 (-11.96,6.73)	0.76 (-9.29,10.81)	4.76 (-7.96,17.49)	0.92 (-9.03,10.86)	3.9 (-5.73,13.52)	8.36 (-4.28,21)	-8.64 (-17.51,0.24)

Expressive Communication	Male	-6.57 (-18.54,5.39)	-0.49 (-15.37,14.39)	9.2 (-5.2,23.6)	4.02 (-11.23,19.26)	2.09 (-12.67,16.85)	-13.65 (-27.14,-0.15)	5.46 (-12.03,22.95)	3.82 (-8.75,16.38)	1.06 (-14.63,16.75)	6.55 (-10.84,23.95)
**Motor Domain**	All	0.01 (-2.05,2.08)	-0.87 (-3.1,1.36)	0.59 (-1.84,3.02)	-0.01 (-2.27,2.24)	-0.01 (-2.3,2.27)	-2.44 (-4.89,0.01)	0.18 (-2.13,2.49)	0.63 (-1.5,2.75)	0.88 (-1.84,3.59)	-0.78 (-3.06,1.5)
**Motor Domain**	Female	1.3 (-1.48,4.07)	-2.08 (-4.75,0.6)	-0.35 (-4.07,3.36)	-1.82 (-4.45,0.81)	-1.88 (-4.74,0.97)	0.12 (-3.55,3.8)	-0.27 (-3.14,2.6)	0.7 (-2.09,3.49)	1.74 (-2.12,5.59)	0.18 (-2.87,3.24)
**Motor Domain**	Male	-0.81 (-4,2.39)	1.82 (-2.07,5.7)	1.87 (-1.92,5.66)	1.92 (-2.07,5.92)	3.9 (0.19,7.61)	-3.02 (-6.63,0.6)	0.93 (-3.7,5.56)	1.35 (-1.95,4.66)	0.82 (-3.32,4.95)	-3.85 (-8.2,0.5)
Fine Motor	All	0.14 (-4.9,5.2)	-2.97 (-8.42,2.48)	2.23 (-3.09,7.54)	-2.98 (-8.46,2.5)	-2.56 (-8.08,2.97)	-5.35 (-11.37,0.67)	-0.48 (-6.13,5.16)	3.18 (-1.97,8.33)	1.46 (-5,7.93)	-1.44 (-6.45,3.57)
Fine Motor	Female	4.18 (-3.67,12.03)	-5.57 (-13.17,2.04)	3.49 (-5.74,12.72)	-6.85 (-14.17,0.48)	-7.14 (-15.02,0.73)	0.2 (-10.22,10.64)	-0.42 (-8.52,7.68)	5.03 (-2.7,12.77)	4.18 (-6.25,14.6)	-0.6 (-8.26,7.07)
Fine Motor	Male	-1.99 (-8.74,4.76)	3.16 (-5.1,11.4)	2.78 (-4.43,9.97)	1.53 (-6.99,10.06)	6.03 (-2,14.07)	-7 (-14.6,0.59)	-0.34 (-10.15,9.47)	3.02 (-3.97,10)	2.26 (-6.47,11)	-5.43 (-13.83,2.98)
Gross Motor	All	-0.26 (-4.57,4.03)	-0.21 (-4.87,4.46)	0.1 (-5.24,5.44)	2.83 (-1.82,7.48)	2.82 (-1.89,7.53)	-3.82 (-8.97,1.32)	1.34 (-3.46,6.14)	-1.15 (-5.57,3.28)	0.36 (-5.31,6.02)	-1.53 (-6.54,3.48)
Gross Motor	Female	0.56 (-5.5,6.63)	-1.79 (-7.71,4.13)	-4.12 (-12.53,4.29)	0.02 (-5.79,5.84)	0.99 (-5.31,7.28)	0.39 (-7.55,8.33)	0.02 (-6.17,6.22)	-2.69 (-8.69,3.29)	-0.24 (-8.64,8.17)	0.97 (-6.05,7.99)
Gross Motor	Male	-0.9 (-7.42,5.62)	3.43 (-4.5,11.35)	3.98 (-4.07,12.04)	5.27 (-2.78,13.32)	8.08 (0.56,15.61)	-4.3 (-11.77,3.2)	3.55 (-5.83,12.93)	1.95 (-4.8,8.71)	0.85 (-7.58,9.27)	-8.54 (-17.75,0.66)
**Cognitive Domain**	All	-0.93 (-3.81,1.95)	-3.4 (-6.45,-0.34)	-0.76 (-3.29,1.76)	-0.55 (-3.7,2.6)	-2.43 (-5.57,0.72)	-1.38 (-4.87,2.1)	-1.68 (-4.89,1.53)	0.75 (-2.24,3.74)	-1.05 (-4.76,2.66)	-2.79 (-5.06,-0.53)
**Cognitive Domain**	Female	0.65 (-2.6,3.9)	-2.7 (-5.78,0.39)	0.16 (-2.66,2.97)	-2.25 (-5.3,0.79)	-2.32 (-5.59,0.95)	0.72 (-3.54,4.99)	-0.99 (-4.29,2.31)	1 (-2.22,4.22)	1.24 (-3.04,5.52)	-1.93 (-4.14,0.28)
**Cognitive Domain**	Male	-1.08 (-5.84,3.68)	-3.13 (-8.87,2.61)	-0.15 (-4.43,4.14)	0.43 (-5.56,6.42)	-1.41 (-7.17,4.35)	-0.73 (-6.22,4.76)	-2.03 (-9.03,4.97)	1.94 (-3.1,6.98)	-1.26 (-7.52,5)	-6.05 (-10.88,-1.22)
Attention and Memory	All	-2.51 (-8.24,3.22)	-6.23 (-12.34,-0.12)	1.15 (-3.22,5.52)	-2.63 (-8.89,3.65)	-4.35 (-10.64,1.94)	-2.82 (-9.77,4.12)	-3.65 (-10.05,2.76)	0.44 (-5.54,6.41)	-0.85 (-8.27,6.57)	-4.52 (-8.46,-0.58)
Attention and Memory	Female	0.26 (-6.74,7.25)	-4.24 (-10.97,2.48)	1.99 (-3.34,7.32)	-4.09 (-10.67,2.49)	-3.4 (-10.51,3.7)	3.88 (-5.2,12.96)	-3.03 (-10.08,4.03)	2.07 (-4.83,8.98)	5.69 (-3.37,14.74)	-4.19 (-8.34,-0.02)
Attention and Memory	Male	-3.37 (-12.15,5.4)	-7.62 (-18.12,2.9)	1.18 (-6.15,8.52)	-4.44 (-15.44,6.55)	-4.64 (-15.25,5.95)	-3.72 (-13.83,6.4)	-4.74 (-17.66,8.18)	0.47 (-8.93,9.87)	-4.64 (-16.17,6.88)	-9.39 (-17.84,-0.94)
Perception and Concepts	All	-0.18 (-4.29,3.92)	-3.69 (-8.08,0.7)	-3.24 (-7.78,1.29)	0.92 (-3.56,5.4)	-2.72 (-7.22,1.77)	-1.13 (-6.1,3.84)	-1.32 (-5.91,3.27)	1.58 (-2.67,5.83)	-2.27 (-7.54,3)	-3.55 (-7.77,0.66)
Perception and Concepts	Female	1.65 (-3.29,6.6)	-3.49 (-8.24,1.27)	-1.36 (-6.57,3.85)	-2.41 (-7.12,2.3)	-3.13 (-8.15,1.88)	-1.63 (-8.12,4.86)	0.09 (-4.97,5.15)	0.8 (-4.13,5.73)	-2.02 (-8.56,4.51)	-1.5 (-5.78,2.77)
Perception and Concepts	Male	0.19 (-6.9,7.28)	-1.84 (-10.46,6.8)	-1.71 (-9.4,5.98)	5.22 (-3.51,13.96)	0.09 (-8.49,8.67)	1.65 (-6.5,9.81)	-1.48 (-11.89,8.95)	4.81 (-2.58,12.19)	0.69 (-8.62,10.01)	-8 (-17.13,1.15)

Table S4. b: Percent change in BDI-2 scores at 12 months per IQR increase in the geometric mean of maternal urinary phthalate metabolite concentrations (ng/ml) across pregnancy (n all = 130, n girls=64, n boys=66).

Domain/Subdomain	sex	MBZP	MCOP	MONP	MCNP	MCPP	MEP	DEHP	DBP	DIBP	DEHTP
**Adaptive Domain**	All	0.73 (-1.56,3.03)	-1.98 (-4.49,0.53)	-0.36 (-2.45,1.73)	-0.04 (-2.19,2.11)	-0.53 (-3.35,2.29)	-2.63 (-4.75,-0.52)	-1.45 (-3.91,1)	-0.16 (-2.33,2.01)	0.11 (-2.57,2.78)	-0.45 (-2.19,1.28)
**Adaptive Domain**	Female	-1.33 (-4.56,1.91)	-1.77 (-5.54,2)	0.11 (-2.45,2.67)	0.87 (-1.97,3.72)	-0.51 (-4.11,3.1)	-2.56 (-5.53,0.41)	-3.46 (-6.65,-0.28)	-2.66 (-5.64,0.33)	-2.21 (-6.07,1.65)	1.26 (-0.58,3.1)
**Adaptive Domain**	Male	2.23 (-1.03,5.48)	-3.67 (-7.26,-0.09)	-1.6 (-5.46,2.26)	-2.34 (-5.71,1.02)	-1.18 (-5.81,3.45)	-3.11 (-6.18,-0.04)	0.44 (-3.33,4.21)	2.25 (-0.83,5.34)	1.73 (-2.03,5.49)	-2.94 (-6.55,0.67)
Self-Care	All	1.4 (-2.99,5.79)	-3.78 (-8.57,1.01)	-0.69 (-4.68,3.31)	-0.07 (-4.18,4.03)	-1.01 (-6.4,4.37)	-5.03 (-9.07,-0.99)	-2.78 (-7.46,1.92)	-0.3 (-4.44,3.85)	0.21 (-4.9,5.31)	-0.87 (-4.18,2.44)
Self-Care	Female	-2.53 (-8.71,3.64)	-3.37 (-10.57,3.82)	0.21 (-4.68,5.1)	1.67 (-3.76,7.1)	-0.97 (-7.85,5.91)	-4.89 (-10.55,0.78)	-6.6 (-12.69,-0.53)	-5.07 (-10.78,0.64)	-4.22 (-11.59,3.15)	2.4 (-1.11,5.92)
Self-Care	Male	4.25 (-1.97,10.47)	-7.02 (-13.87,-0.16)	-3.06 (-10.42,4.32)	-4.48 (-10.9,1.95)	-2.26 (-11.1,6.58)	-5.95 (-11.8,-0.08)	0.83 (-6.37,8.04)	4.31 (-1.59,10.19)	3.31 (-3.88,10.48)	-5.61 (-12.5,1.28)
**Personal-Social Domain**	All	-1.16 (-2.99,0.68)	-3.28 (-5.25,-1.3)	-1.21 (-3.06,0.65)	-2.09 (-3.76,-0.43)	-1.09 (-3.33,1.14)	-0.61 (-2.37,1.16)	-1.11 (-3.11,0.88)	-0.63 (-2.37,1.11)	-0.33 (-2.45,1.79)	0.01 (-1.53,1.56)
**Personal-Social Domain**	Female	-1.58 (-4.53,1.36)	-4.03 (-7.31,-0.75)	-2.17 (-5.23,0.89)	-2.03 (-4.56,0.5)	-1.41 (-4.66,1.85)	-1.95 (-4.66,0.77)	-3.66 (-6.64,-0.68)	-2.52 (-5.3,0.26)	-1.23 (-4.77,2.3)	-0.56 (-2.82,1.7)
**Personal-Social Domain**	Male	-0.83 (-3.23,1.57)	-3.45 (-6.13,-0.77)	-0.49 (-3.13,2.15)	-2.35 (-4.76,0.05)	-0.53 (-3.89,2.82)	0.71 (-1.71,3.12)	1.52 (-1.19,4.22)	0.97 (-1.27,3.22)	0.4 (-2.32,3.13)	0.63 (-1.89,3.15)
Adult Interaction	All	-2.69 (-6.74,1.37)	-7.23 (-11.62,-2.83)	-3.53 (-7.66,0.62)	-4.64 (-8.35,-0.94)	-4.11 (-9.04,0.81)	-0.12 (-4.05,3.82)	-2.47 (-6.82,1.88)	-1.01 (-4.82,2.8)	-0.74 (-5.44,3.95)	-1.72 (-5.19,1.73)
Adult Interaction	Female	-4.76 (-11,1.48)	-7.31 (-14.48,-0.13)	-4.8 (-11.53,1.94)	-3.82 (-9.31,1.68)	-5.92 (-12.8,0.97)	-1.2 (-7.16,4.78)	-6.4 (-12.67,-0.13)	-3.85 (-9.78,2.09)	-3.46 (-11.06,4.13)	-1.65 (-6.65,3.36)
Adult Interaction	Male	-0.57 (-6.12,4.98)	-7.97 (-14.15,-1.8)	-2.26 (-8.22,3.7)	-5.66 (-11.18,-0.14)	-0.84 (-8.58,6.9)	0.51 (-5.07,6.09)	2.55 (-3.71,8.81)	1.57 (-3.57,6.72)	1.67 (-4.53,7.88)	-3.47 (-9.1,2.16)
Self-Concept and Social Role	All	-1.83 (-6.58,2.93)	-5.88 (-11.02,-0.75)	-1.24 (-6.17,3.69)	-3.91 (-8.29,0.48)	-0.17 (-6.01,5.66)	-2.51 (-6.97,1.95)	-2.15 (-7.34,3.05)	-1.57 (-6.11,2.96)	-0.48 (-6,5.06)	1.86 (-2.19,5.91)
Self-Concept and Social Role	Female	-1.45 (-9.09,6.19)	-9.3 (-17.83,-0.78)	-3.88 (-11.61,3.86)	-4.68 (-11.22,1.86)	0.37 (-8.07,8.8)	-7.07 (-13.93,-0.23)	-8.29 (-16.04,-0.54)	-6.28 (-13.44,0.88)	-1.27 (-10.4,7.85)	-0.59 (-6.25,5.08)
Self-Concept and Social Role	Male	-2.79 (-8.98,3.39)	-5.08 (-11.95,1.79)	0.4 (-7.05,7.86)	-3.7 (-10.07,2.67)	-1.26 (-10,7.47)	2.33 (-3.63,8.28)	3.51 (-3.54,10.55)	2.36 (-3.52,8.25)	-0.03 (-7.17,7.11)	6.32 (-0.55,13.2)
**Communication Domain**	All	0.83 (-1.79,3.45)	0.83 (-2.05,3.72)	1.21 (-1.41,3.84)	1.61 (-0.82,4.04)	0.85 (-2.36,4.06)	-0.81 (-3.28,1.65)	2.34 (-0.45,5.12)	0.44 (-2.03,2.92)	0.35 (-2.7,3.4)	-0.13 (-2.31,2.06)
**Communication Domain**	Female	-0.01 (-3.87,3.85)	1.53 (-2.96,6.02)	1.18 (-2.92,5.28)	1.68 (-1.68,5.03)	1.19 (-3.08,5.46)	-0.34 (-3.96,3.28)	1 (-2.93,4.93)	-0.36 (-4,3.29)	-1.84 (-6.45,2.76)	1.25 (-1.74,4.23)
**Communication Domain**	Male	1.87 (-1.81,5.55)	0.17 (-4,4.35)	2.11 (-1.89,6.11)	1.69 (-2.12,5.51)	0.73 (-4.48,5.94)	-1.81 (-5.34,1.73)	4.71 (0.66,8.76)	1.15 (-2.36,4.67)	1.99 (-2.23,6.21)	-2.66 (-6.44,1.12)
Receptive Communication	All	1.58 (-5.59,8.74)	-4.17 (-12.03,3.68)	0.12 (-7.06,7.31)	2.38 (-4.3,9.05)	-1.2 (-9.99,7.57)	-1.28 (-8.03,5.48)	1.76 (-5.92,9.44)	3.15 (-3.59,9.89)	5.49 (-2.79,13.75)	-0.5 (-6.46,5.46)
Receptive Communication	Female	-0.13 (-10.58,10.31)	3.54 (-8.62,15.7)	4.13 (-6.65,14.93)	5.16 (-3.9,14.21)	-0.14 (-11.72,11.44)	-3.48 (-13.22,6.27)	-2.7 (-13.34,7.94)	-2.97 (-12.8,6.87)	0.39 (-12.15,12.92)	2.75 (-5.15,10.66)
Receptive Communication	Male	4.37 (-5.56,14.3)	-8.62 (-19.61,2.38)	-1.73 (-12.67,9.2)	1.11 (-9.21,11.44)	0.8 (-13.22,14.81)	-0.76 (-10.35,8.84)	9.77 (-1.33,20.88)	9.18 (0,18.36)	10.82 (-0.26,21.9)	-8.52 (-18.68,1.63)
Expressive Communication	All	1.71 (-3.9,7.31)	5.82 (-0.27,11.92)	4.01 (-1.63,9.65)	3.75 (-1.46,8.95)	3.76 (-3.1,10.61)	-1.88 (-7.17,3.41)	6.55 (0.63,12.46)	-0.79 (-6.08,4.51)	-2.88 (-9.38,3.62)	-0.11 (-4.83,4.61)
Expressive Communication	Female	0.12 (-8.14,8.38)	2.53 (-7.09,12.15)	1 (-7.88,9.89)	1.91 (-5.32,9.14)	4.15 (-4.94,13.23)	1.11 (-6.63,8.85)	5.22 (-3.09,13.53)	0.9 (-6.9,8.71)	-6.59 (-16.35,3.16)	2.27 (-4.21,8.74)
Expressive Communication	Male	3.14 (-4.84,11.13)	6.87 (-1.98,15.71)	8.4 (-0.09,16.89)	4.94 (-3.26,13.15)	1.84 (-9.42,13.1)	-5.41 (-12.99,2.17)	8.69 (-0.18,17.57)	-2.68 (-10.27,4.91)	-1.16 (-10.35,8.01)	-2.89 (-11.27,5.49)

**Motor Domain**	All	-0.66 (-2.67,1.36)	-1.82 (-4.02,0.37)	-0.9 (-2.87,1.07)	-1.16 (-3.04,0.71)	-1.02 (-3.48,1.44)	-0.56 (-2.46,1.34)	-0.88 (-3.04,1.28)	-0.53 (-2.43,1.37)	0.28 (-2.06,2.63)	1.58 (-0.03,3.19)
**Motor Domain**	Female	0.5 (-2.47,3.47)	0.59 (-2.87,4.06)	0.35 (-2.71,3.41)	0.15 (-2.45,2.76)	-0.21 (-3.51,3.08)	-0.67 (-3.45,2.11)	-0.82 (-3.85,2.2)	-0.43 (-3.24,2.37)	2.59 (-0.91,6.09)	1.27 (-0.94,3.49)
**Motor Domain**	Male	-1.71 (-4.5,1.09)	-3.99 (-7,-0.99)	-1.94 (-4.86,0.99)	-2.72 (-5.56,0.12)	-1.58 (-5.54,2.37)	-0.55 (-3.27,2.17)	-0.61 (-3.84,2.62)	-0.47 (-3.15,2.22)	-1.3 (-4.51,1.92)	1.98 (-0.8,4.76)
Fine Motor	All	2.09 (-2.22,6.4)	-3.66 (-8.37,1.05)	0.16 (-3.58,3.89)	-2.79 (-6.8,1.21)	1.04 (-4.26,6.34)	0.15 (-3.93,4.23)	0.27 (-4.38,4.91)	2.8 (-1.25,6.85)	0.32 (-4.72,5.34)	2.45 (-0.61,5.52)
Fine Motor	Female	3.42 (-2.72,9.54)	0.78 (-6.45,8.03)	3.86 (-2.26,9.98)	-0.88 (-6.32,4.55)	1.6 (-5.26,8.45)	-0.05 (-5.86,5.77)	-1.87 (-8.17,4.44)	1.98 (-3.85,7.81)	1.95 (-5.47,9.37)	3.43 (-1,7.88)
Fine Motor	Male	0.94 (-5.37,7.25)	-7.52 (-14.33,-0.71)	-0.95 (-5.84,3.93)	-4.66 (-11.07,1.75)	1.49 (-7.35,10.34)	0.55 (-5.5,6.61)	3.21 (-3.95,10.36)	3.72 (-2.19,9.63)	-0.6 (-7.82,6.61)	0.6 (-4.07,5.26)
Gross Motor	All	-6.05 (-12.2,0.1)	-3.53 (-10.37,3.31)	-4.4 (-11.12,2.31)	-1.79 (-7.61,4.03)	-6.01 (-13.57,1.56)	-2.87 (-8.73,2.99)	-4.53 (-11.18,2.12)	-6.32 (-12.09,-0.56)	0.8 (-6.45,8.06)	4.03 (-1.53,9.58)
Gross Motor	Female	-2.56 (-11.88,6.76)	1.26 (-9.64,12.17)	-3.77 (-13.64,6.08)	1.66 (-6.53,9.84)	-2.96 (-13.28,7.37)	-3.11 (-11.82,5.62)	-1.56 (-11.09,7.97)	-4.73 (-13.46,4)	9.01 (-1.94,19.96)	1.18 (-6.07,8.43)
Gross Motor	Male	-9.25 (-17.67,-0.81)	-8.03 (-17.65,1.61)	-7.49 (-18.43,3.45)	-6.39 (-15.3,2.53)	-9.35 (-21.42,2.7)	-3.39 (-11.77,4.99)	-7.05 (-16.88,2.8)	-7.33 (-15.42,0.77)	-5.11 (-15.04,4.83)	8.64 (-1.7,18.99)
**Cognitive Domain**	All	0.52 (-1.36,2.4)	-2.61 (-4.63,-0.6)	-1.05 (-2.91,0.81)	-0.96 (-2.71,0.79)	-1 (-3.3,1.29)	-1.2 (-2.96,0.56)	-1 (-3.01,1.01)	0.53 (-1.23,2.3)	0.02 (-2.16,2.2)	-0.78 (-2.33,0.77)
**Cognitive Domain**	Female	1.5 (-1.08,4.08)	-2.28 (-5.27,0.71)	-0.2 (-2.65,2.26)	-1.22 (-3.49,1.04)	-0.39 (-3.28,2.51)	-0.64 (-3.08,1.8)	-0.97 (-3.62,1.69)	-0.65 (-3.11,1.81)	0.08 (-3.05,3.22)	-0.09 (-1.89,1.7)
**Cognitive Domain**	Male	-0.03 (-2.75,2.69)	-3.04 (-5.99,-0.09)	-1.62 (-4.71,1.48)	-0.67 (-3.47,2.14)	-1.26 (-5.06,2.54)	-2.34 (-4.88,0.2)	-0.3 (-3.4,2.81)	1.81 (-0.71,4.33)	0.16 (-2.93,3.26)	-2.1 (-5.02,0.82)
Attention and Memory	All	1.94 (-2.11,6.01)	-8.23 (-12.45,-4)	-1.81 (-5.92,2.3)	-4.44 (-8.16,-0.72)	-0.87 (-5.87,4.13)	-2.57 (-6.37,1.25)	-2.79 (-7.13,1.55)	2.67 (-1.13,6.46)	0.72 (-3.99,5.44)	-1.68 (-5.09,1.72)
Attention and Memory	Female	2.96 (-2.63,8.56)	-8.91 (-15.05,-2.77)	0.32 (-5.1,5.74)	-6 (-10.69,-1.32)	-1.49 (-7.74,4.76)	-1.83 (-7.1,3.45)	-4.4 (-10.04,1.24)	-0.59 (-5.92,4.75)	-0.49 (-7.26,6.29)	-0.39 (-4.35,3.58)
Attention and Memory	Male	1.72 (-4.34,7.79)	-7.61 (-14.14,-1.08)	-2.09 (-9.23,5.05)	-2.15 (-8.41,4.11)	1.38 (-7.14,9.91)	-4.02 (-9.77,1.73)	0.03 (-6.92,6.96)	5.89 (0.37,11.41)	2.25 (-4.64,9.14)	-3.51 (-10.27,3.25)
Perception and Concepts	All	-0.63 (-3.55,2.29)	0.88 (-2.33,4.1)	-1.38 (-4.45,1.69)	1.81 (-0.89,4.53)	-2.27 (-5.84,1.28)	-0.66 (-3.43,2.09)	-0.1 (-3.24,3.03)	-1.21 (-3.95,1.54)	-0.58 (-3.98,2.82)	-0.69 (-3.25,1.86)
Perception and Concepts	Female	1.36 (-2.89,5.61)	2.63 (-2.3,7.55)	-0.99 (-5.27,3.27)	2.72 (-0.94,6.39)	0.17 (-4.56,4.9)	0.23 (-3.76,4.23)	1.74 (-2.59,6.07)	-1.28 (-5.3,2.73)	0.95 (-4.16,6.06)	-0.01 (-3.13,3.12)
Perception and Concepts	Male	-2.13 (-6.01,1.74)	-1.05 (-5.44,3.34)	-2.91 (-7.69,1.88)	0.22 (-3.82,4.27)	-5.31 (-10.61,0)	-2.61 (-6.3,1.07)	-0.97 (-5.43,3.49)	-0.8 (-4.51,2.91)	-1.78 (-6.23,2.69)	-2.77 (-7.32,1.8)

Table S4. c: Percent change in BDI-2 scores at 24 months per IQR increase in the geometric mean of maternal urinary phthalate metabolite concentrations (ng/ml) across pregnancy (n all = 123, n girls=69, n boys=54).

Domain/Subdomain	sex	MBZP	MCOP	MONP	MCNP	MCPP	MEP	DEHP	DBP	DIBP	DEHTP
**Adaptive Domain**	All	-2.01 (-5.05,1.03)	-2.81 (-5.43,-0.19)	-4.04 (-7.62,-0.45)	-3.36 (-6.25,-0.46)	-4.95 (-7.7,-2.2)	1.28 (-2.04,4.6)	-1.61 (-4.01,0.79)	-4.77 (-7.77,-1.77)	-0.25 (-3.61,3.1)	-1.71 (-4.86,1.44)
**Adaptive Domain**	Female	-4.3 (-7.92,-0.68)	-2.11 (-5.01,0.78)	-2.01 (-6.88,2.87)	-2.03 (-5.12,1.06)	-3.8 (-6.87,-0.72)	1.29 (-2.34,4.92)	-1.87 (-4.8,1.07)	-3.16 (-7.35,1.04)	0.38 (-3.56,4.32)	-2.24 (-6.06,1.58)
**Adaptive Domain**	Male	0.34 (-5.48,6.16)	-5.55 (-11.16,0.06)	-5.94 (-11.63,-0.25)	-9.09 (-15.92,-2.26)	-9.84 (-15.74,-3.94)	1.08 (-6.22,8.39)	-1.57 (-6.8,3.65)	-6.95 (-11.79,-2.12)	-1.77 (-8.51,4.96)	-1.88 (-8.1,4.34)
Self-Care	All	-1.76 (-9.07,5.57)	1.32 (-5.11,7.77)	-3.46 (-11.22,4.28)	0.83 (-6.19,7.83)	-4.73 (-11.73,2.27)	1.26 (-6.78,9.3)	-2.23 (-8.05,3.59)	-7.05 (-14.55,0.47)	2.12 (-6.01,10.24)	-1.12 (-7.81,5.59)
Self-Care	Female	-7.65 (-17.38,2.08)	4.39 (-3.23,12)	3.18 (-7.11,13.47)	2.77 (-5.39,10.95)	-2.8 (-11.26,5.66)	-0.31 (-9.84,9.21)	-4.91 (-12.57,2.75)	-4.02 (-15.17,7.12)	2.52 (-7.75,12.8)	0.54 (-7.66,8.73)
Self-Care	Male	3.14 (-8.91,15.19)	-8.36 (-20.68,3.95)	-9.45 (-21.35,2.45)	-7.88 (-22.24,6.47)	-15.07 (-28.68,-1.46)	4.47 (-11.1,20.04)	1.81 (-9.29,12.91)	-10.68 (-21.73,0.37)	0.74 (-13.82,15.28)	-4.6 (-17.33,8.12)
**Personal-Social Domain**	All	-0.23 (-3.37,2.91)	-3.15 (-5.81,-0.48)	-5.09 (-8.95,-1.24)	-1.45 (-4.39,1.49)	-5.42 (-8.15,-2.69)	1.31 (-2.13,4.75)	-1.36 (-3.83,1.11)	-2.16 (-5.35,1.03)	0.58 (-2.91,4.07)	-0.02 (-3.38,3.34)
**Personal-Social Domain**	Female	-2.14 (-5.59,1.3)	-0.76 (-3.38,1.85)	-2.29 (-6.94,2.35)	0.12 (-2.62,2.85)	-3.6 (-6.27,-0.94)	0.1 (-3.21,3.42)	-1.48 (-4.09,1.13)	-1.09 (-4.85,2.67)	-0.48 (-3.98,3.02)	0.33 (-3.08,3.75)
**Personal-Social Domain**	Male	2.09 (-3.97,8.15)	-8.39 (-14.19,-2.59)	-7.6 (-13.56,-1.63)	-5.43 (-12.54,1.68)	-9.84 (-16.38,-3.3)	2.52 (-5.29,10.32)	0.15 (-5.62,5.92)	-3.76 (-9.54,2.02)	1.42 (-6.18,9.03)	-1.97 (-8.73,4.8)
Adult Interaction	All	-1.69 (-9.75,6.38)	-8.31 (-15.21,-1.41)	-11.77 (-21.54,-1.99)	-4.36 (-12.03,3.31)	-14.06 (-21.29,-6.84)	1.83 (-7.07,10.73)	-4.06 (-10.43,2.3)	-5.23 (-13.56,3.11)	-3.05 (-11.96,5.87)	1.92 (-6.51,10.35)
Adult Interaction	Female	-4.75 (-15.11,5.62)	-4.63 (-12.61,3.35)	-6.27 (-19.37,6.84)	-1.81 (-10.33,6.72)	-10.79 (-19.16,-2.42)	-1.11 (-11.16,8.91)	-4.14 (-12.16,3.89)	-6.13 (-17.65,5.39)	-9.46 (-19.86,0.93)	2.37 (-7.22,11.96)
Adult Interaction	Male	3.01 (-11.2,17.22)	-17.44 (-31.11,-3.77)	-17.18 (-31.5,-2.86)	-11.63 (-28.37,5.12)	-22.49 (-37.82,-7.15)	4.69 (-13.69,23.05)	-1.65 (-14.74,11.44)	-6.6 (-20.09,6.9)	3.83 (-13.22,20.88)	-1.52 (-17.72,14.67)
Self-Concept and Social Role	All	-4.09 (-10.21,2.04)	-6.41 (-11.6,-1.22)	-11.8 (-18.84,-4.76)	-2.87 (-8.69,2.94)	-10.15 (-15.62,-4.67)	-1.21 (-7.97,5.57)	-4.31 (-9.1,0.48)	-7.38 (-13.54,-1.22)	-1.19 (-7.97,5.6)	0.52 (-6.02,7.05)
Self-Concept and Social Role	Female	-11.75 (-19.15,-4.34)	-3.01 (-8.97,2.95)	-9.59 (-19.48,0.3)	-1.09 (-7.47,5.27)	-7.07 (-13.41,-0.73)	-2.69 (-10.3,4.92)	-6.82 (-12.69,-0.95)	-7.32 (-15.86,1.23)	-0.06 (-8.22,8.1)	-1.05 (-9.33,7.23)
Self-Concept and Social Role	Male	3.86 (-7.01,14.71)	-13.6 (-24.06,-3.13)	-14.54 (-25.15,-3.92)	-7.79 (-20.79,5.22)	-17.04 (-28.84,-5.25)	-1.4 (-15.57,12.76)	1.26 (-8.79,11.32)	-8.94 (-19.09,1.21)	-4.54 (-17.72,8.65)	0.05 (-12.22,12.32)
**Communication Domain**	All	-1.71 (-7,3.58)	-7.67 (-12.06,-3.29)	-9.86 (-15.71,-4)	-3.84 (-8.85,1.17)	-10.67 (-15.28,-6.06)	1.05 (-4.76,6.86)	-4.16 (-8.3,-0.03)	-5.74 (-11.15,-0.33)	-0.44 (-6.32,5.45)	-4.41 (-9.73,0.92)
**Communication Domain**	Female	-3.61 (-10.43,3.2)	-4.79 (-9.96,0.38)	-5.02 (-13.8,3.76)	-1.89 (-7.54,3.76)	-8.34 (-13.74,-2.94)	-0.66 (-7.25,5.93)	-5.59 (-10.75,-0.43)	-6.41 (-13.96,1.13)	0.32 (-6.81,7.44)	-4.21 (-11.15,2.73)
**Communication Domain**	Male	1.32 (-8.12,10.77)	-13.27 (-22.07,-4.47)	-13.96 (-22.34,-5.57)	-8.16 (-19.23,2.92)	-15.82 (-25.84,-5.8)	3.22 (-8.97,15.41)	-0.48 (-9.17,8.21)	-5.09 (-14.02,3.84)	-1.16 (-12.52,10.2)	-6.01 (-15.94,3.92)
Receptive Communication	All	-6.72 (-18.23,4.77)	-17.81 (-27.31,-8.31)	-18.78 (-31.79,-5.77)	-7.05 (-18.05,3.93)	-21.28 (-31.55,-11.01)	3.15 (-9.53,15.84)	-11.55 (-20.47,-2.63)	-14.36 (-26.08,-2.64)	-3.19 (-16.02,9.64)	-11.63 (-23.14,-0.12)
Receptive Communication	Female	-9.9 (-24.45,4.65)	-8.43 (-19.67,2.8)	-0.02 (-18.31,18.27)	-1.14 (-13.33,11.06)	-14.42 (-26.41,-2.43)	-1.49 (-15.67,12.67)	-12.89 (-23.9,-1.88)	-15.13 (-31.25,0.99)	1.56 (-13.76,16.88)	-5.38 (-19.75,8.99)
Receptive Communication	Male	-3.84 (-24.79,17.09)	-39.02 (-56.52,-21.53)	-33.55 (-52.15,-14.94)	-23.19 (-47.26,0.89)	-41.4 (-62.35,-20.44)	11.88 (-14.96,38.73)	-10.1 (-29.08,8.89)	-13.17 (-32.86,6.52)	-9.1 (-34.16,15.94)	-20.94 (-42.71,0.85)
Expressive Communication	All	-0.38 (-14.08,13.31)	-15.41 (-27.03,-3.8)	-24.4 (-39.67,-9.13)	-9.27 (-22.24,3.7)	-24.37 (-36.57,-12.15)	0.23 (-14.78,15.26)	-6.04 (-16.88,4.79)	-10.97 (-25.11,3.17)	0.54 (-14.67,15.75)	-6.51 (-20.49,7.46)

Expressive Communication	Female	-5.32 (-23.08,12.46)	-12.26 (-25.65,1.13)	-22.52 (-45.05,0)	-6.96 (-21.52,7.61)	-21.01 (-35.06,-6.95)	-1.85 (-18.91,15.22)	-11.16 (-24.76,2.43)	-12.32 (-32.09,7.44)	-1.37 (-19.8,17.07)	-12.43 (-30.84,5.97)
Expressive Communication	Male	9.57 (-14.18,33.34)	-18.49 (-42.74,5.76)	-27.17 (-49.44,-4.9)	-11.23 (-39.92,17.46)	-26.87 (-54.2,0.46)	-0.5 (-31.53,30.52)	8.49 (-13.37,30.34)	-10.12 (-32.91,12.67)	3.84 (-24.93,32.61)	-3.67 (-28.89,21.56)
**Motor Domain**	All	0.23 (-1.71,2.17)	-2.03 (-3.73,-0.33)	-3.03 (-5.43,-0.63)	-1.21 (-3.15,0.73)	-2.45 (-4.35,-0.55)	-0.61 (-2.75,1.53)	-0.13 (-1.72,1.46)	-1.92 (-3.91,0.06)	-0.2 (-2.31,1.92)	-2.05 (-4.44,0.34)
**Motor Domain**	Female	-1 (-3.44,1.45)	-1.6 (-3.51,0.31)	-1.9 (-5.36,1.57)	-1.32 (-3.46,0.83)	-2.73 (-4.79,-0.67)	-0.75 (-3.12,1.63)	-1.04 (-2.94,0.86)	-2.18 (-4.86,0.51)	-1.15 (-3.66,1.37)	0.61 (-2.72,3.94)
**Motor Domain**	Male	1 (-2.44,4.44)	-4.46 (-7.8,-1.12)	-3.85 (-7.3,-0.4)	-2.36 (-6.63,1.92)	-4.01 (-8.11,0.08)	-0.39 (-4.92,4.13)	0.56 (-2.64,3.76)	-2.79 (-6.04,0.45)	0.32 (-3.76,4.39)	-4.95 (-8.29,-1.61)
Fine Motor	All	-0.51 (-4.81,3.78)	-5.24 (-8.89,-1.59)	-6.96 (-11.97,-1.96)	-3.31 (-7.54,0.91)	-7.09 (-11.07,-3.1)	0 (-4.69,4.7)	-0.47 (-3.99,3.05)	-3.62 (-8,0.74)	-1.39 (-6.09,3.31)	-4.18 (-9.22,0.86)
Fine Motor	Female	-1.05 (-6.68,4.58)	-4.39 (-8.67,-0.12)	-4.58 (-11.81,2.66)	-3.95 (-8.77,0.88)	-6.99 (-11.56,-2.42)	1.08 (-4.3,6.46)	-1.62 (-6.03,2.79)	-3.36 (-9.62,2.9)	-2.76 (-8.53,3.01)	-3.84 (-10.7,3)
Fine Motor	Male	0.68 (-6.96,8.31)	-7.94 (-15.29,-0.6)	-8.53 (-15.89,-1.15)	-1.31 (-10.58,7.96)	-8.76 (-17.39,-0.12)	-1.78 (-11.74,8.2)	0.84 (-6.24,7.92)	-4.67 (-11.52,2.19)	0.37 (-8.49,9.22)	-5.66 (-13.69,2.37)
Gross Motor	All	1.2 (-3.54,5.93)	-1.07 (-5.28,3.15)	-3.65 (-9.27,1.98)	-3.44 (-8.11,1.23)	-1.58 (-6.29,3.14)	-2.13 (-7.34,3.07)	1.07 (-2.74,4.88)	-1.75 (-6.66,3.16)	1.55 (-3.66,6.77)	-1.48 (-7.01,4.05)
Gross Motor	Female	-2.26 (-8.68,4.16)	-0.67 (-5.74,4.42)	-4.41 (-12.13,3.32)	-2.69 (-8.28,2.9)	-3.73 (-9.39,1.93)	-3.83 (-9.97,2.33)	-2.61 (-7.61,2.39)	-2.33 (-9.55,4.87)	-2.1 (-8.72,4.51)	3.49 (-3.83,10.81)
Gross Motor	Male	4.68 (-2.83,12.19)	-4.57 (-12.44,3.3)	-3.47 (-11.83,4.9)	-7.99 (-16.86,0.87)	0.46 (-8.77,9.68)	-0.22 (-10.16,9.71)	8.32 (1.84,14.8)	-2.88 (-10.2,4.44)	5.25 (-3.8,14.31)	-6.47 (-14.91,1.97)
**Cognitive Domain**	All	0.19 (-2.97,3.34)	-3.24 (-5.84,-0.64)	-5.61 (-9.04,-2.18)	-1.65 (-4.59,1.29)	-4.69 (-7.47,-1.9)	-1.58 (-4.81,1.65)	0.29 (-2.08,2.65)	-3.08 (-6.05,-0.12)	-1.98 (-5.22,1.25)	-2.76 (-6.77,1.26)
**Cognitive Domain**	Female	0.59 (-3.24,4.42)	-0.38 (-3.1,2.33)	-1.75 (-6.32,2.82)	0.16 (-2.72,3.04)	-3.1 (-5.94,-0.26)	-1.33 (-4.47,1.81)	-0.71 (-3.31,1.9)	-2.69 (-6.44,1.06)	-1.32 (-4.72,2.09)	-1.45 (-7.17,4.27)
**Cognitive Domain**	Male	0.16 (-5.7,6.03)	-9.85 (-14.89,-4.81)	-8.65 (-13.98,-3.31)	-7.48 (-14.79,-0.18)	-10.22 (-16.32,-4.12)	-1.38 (-9.24,6.48)	1.61 (-4.12,7.33)	-3.44 (-8.64,1.76)	-2.91 (-9.85,4.03)	-3.63 (-10.16,2.89)
Attention and Memory	All	-0.68 (-7.78,6.4)	-5.03 (-11.2,1.15)	-10.55 (-18.43,-2.66)	-1.37 (-8.01,5.28)	-7.13 (-13.74,-0.5)	-3.25 (-10.28,3.78)	-0.69 (-6,4.62)	-6.07 (-12.88,0.72)	-2.6 (-9.88,4.7)	-5.44 (-13.99,3.13)
Attention and Memory	Female	-0.86 (-9.71,7.99)	1.12 (-5.55,7.77)	0.62 (-9.54,10.78)	1.14 (-5.89,8.18)	-3.56 (-10.79,3.67)	-0.82 (-8.21,6.59)	-1.88 (-8.01,4.25)	-3.87 (-12.91,5.17)	-0.07 (-8.29,8.14)	-1.42 (-12.35,9.54)
Attention and Memory	Male	-1.36 (-13.71,11)	-20.2 (-31.48,-8.92)	-18.99 (-30.42,-7.55)	-11.14 (-25.55,3.28)	-20.68 (-33.59,-7.78)	-7.18 (-22.37,8.02)	-0.24 (-11.83,11.34)	-7.65 (-18.78,3.48)	-6.57 (-20.99,7.85)	-8.49 (-22.59,5.6)
Perception and Concepts	All	2.89 (-4.69,10.49)	-7.61 (-14.02,-1.18)	-11.37 (-19.8,-2.95)	-3.16 (-10.3,3.99)	-9.03 (-15.96,-2.1)	-4.72 (-12.45,3)	4.79 (-0.83,10.42)	-5.28 (-12.55,1.99)	-2.78 (-10.55,4.97)	-2.44 (-11.73,6.84)
Perception and Concepts	Female	2.19 (-6.43,10.82)	-0.13 (-6.28,6.03)	-1.22 (-10.5,8.06)	1.59 (-4.87,8.04)	-4.8 (-11.41,1.82)	-1.99 (-9.13,5.16)	1.66 (-4.2,7.52)	-0.76 (-9.42,7.91)	-1.5 (-9.21,6.21)	0.76 (-9.31,10.82)
Perception and Concepts	Male	2.18 (-11.83,16.19)	-22.97 (-35.68,-10.25)	-18.69 (-32.41,-4.97)	-15.58 (-32.7,1.53)	-22.25 (-37.05,-7.45)	-7.85 (-25.59,9.89)	5.01 (-8.16,18.18)	-9.26 (-21.83,3.3)	-5.28 (-21.73,11.19)	-6.17 (-22.54,10.21)

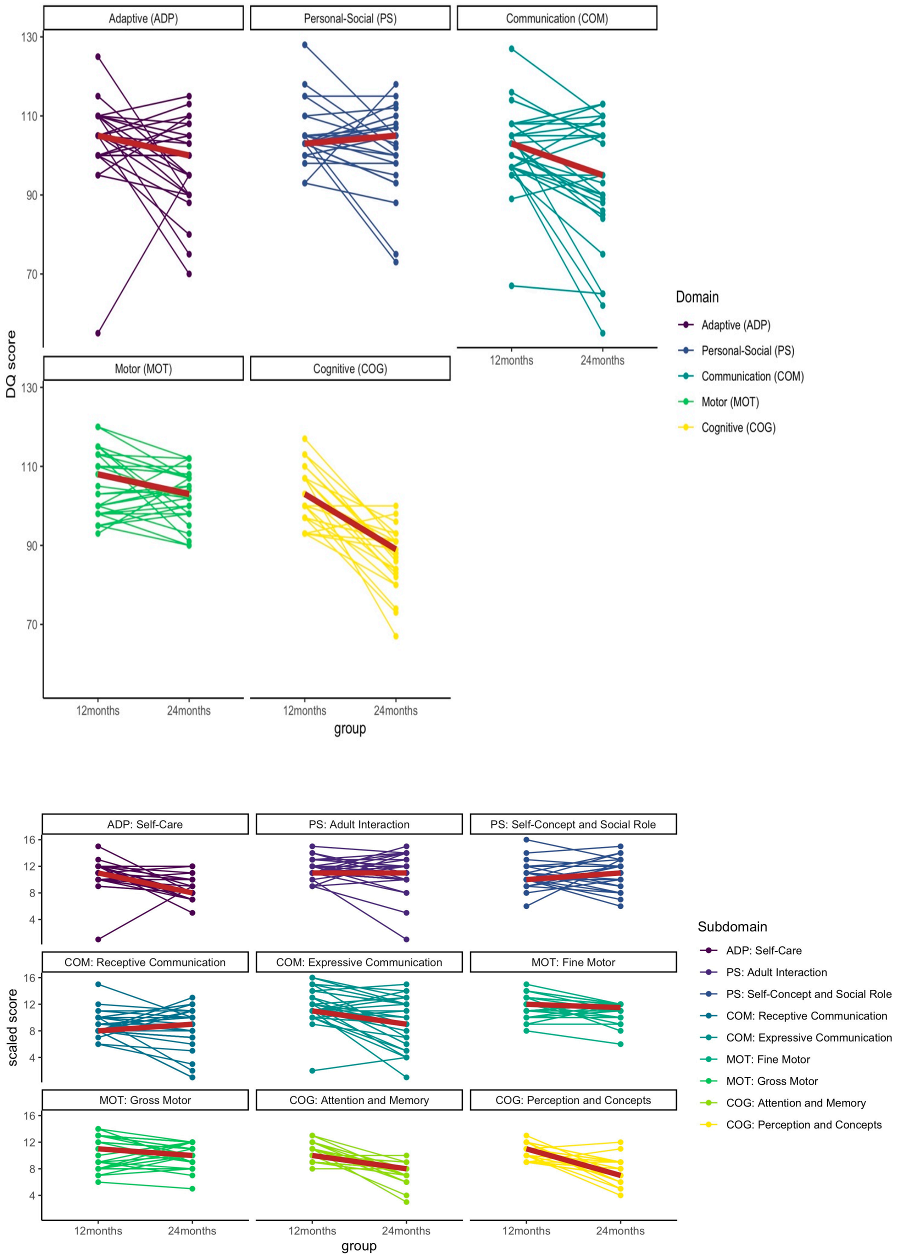

Figure S2. Spaghetti plots of the change of BDI Domain/Subdomain score of children whose score were reported twice (at age 12 months and 24 months) during the study period. Brown solid lines indicate the median change of score.
